# Multimodal EEG-fNIRS complexity analysis can reveal the neurovascular coupling change with aging

**DOI:** 10.3389/fnagi.2026.1912923

**Published:** 2026-10-26

**Authors:** Lingxuan Lv, Ziyue Liu, Wenxin Li, Feifei Zhai, Rui Chen, Yicheng Zhu

**Affiliations:** 1 Department of Neurology, State Key Laboratory of Complex Severe and Rare Diseases, Peking Union Medical College Hospital, Chinese Academy of Medical Sciences and Peking Union Medical College, Beijing, China; 2 Clinical Pharmacology Research Center, Peking Union Medical College Hospital, Chinese Academy of Medical Sciences and Peking Union Medical College, Beijing, China

**Keywords:** aging, complexity analysis, EEG, entropy, fNIRS, neurovascular coupling

## Abstract

**Objective:**

This study aimed to investigate age-related changes in neurovascular coupling (NVC) function of the brain using a multimodal electroencephalography (EEG) and functional near-infrared spectroscopy (fNIRS) approach combined with nonlinear entropy analysis.

**Methods:**

The study included 20 young adults and 20 older adults. We simultaneously recorded EEG and fNIRS data during four cognitive tasks (auditory attention task, verbal fluency task, upper limb motor task, and recognition memory task). We calculated sample entropy (SampEn) and conditional entropy (CondEn) for five EEG frequency sub-bands (Delta: 1–4 Hz, Theta: 4–8 Hz, Alpha: 8–12 Hz, Beta: 12–30 Hz, Gamma: 30–40 Hz) as well as for fNIRS signals (oxygenated hemoglobin and deoxygenated hemoglobin, HbO and HbR) to quantify neural and hemodynamic complexity of brain activity. Additionally, cross-sample entropy (Cross-SampEn) and cross-conditional entropy (Cross-CondEn) between EEG and fNIRS signals were computed to evaluate NVC function. Group differences were assessed using analysis of covariance (ANCOVA), with age group as the fixed factor and sex and years of education as covariates.

**Results:**

Older adults showed higher EEG SampEn or CondEn in selected frequency bands during the auditory attention task and the encoding and recognition phases of the recognition memory task. For fNIRS, older adults showed lower HbO SampEn during memory recognition. Cross-modal analysis showed lower beta-HbR Cross-SampEn during the left upper limb motor task and higher theta-HbR Cross-SampEn during memory encoding in older adults.

**Conclusion:**

Aging is associated with task-specific alterations in neural complexity, hemodynamic complexity, and the cross-modal temporal relationship between EEG and fNIRS signals. These findings suggest that age-related changes in NVC-related dynamics are better characterized as dynamic reorganization rather than a uniform enhancement or impairment, and support the potential of multimodal entropy analysis for characterizing age-related neurovascular coupling change.

## 1 Introduction

Neurovascular Coupling (NVC) refers to the dynamic matching process between neuronal activity and regional cerebral blood flow (CBF) (Owens et al., 2024). This coupling mechanism is crucial for maintaining normal brain function, as it ensures that neurons receive adequate energy supply during various cognitive activities (Dion-Albert et al., 2023).

As an inevitable biological process, aging is typically accompanied by tissue atrophy and functional decline, with the brain being one of the most significantly affected organs (Isaev et al., 2019). The aging process not only leads to cerebral atrophy but also alters NVC function (Tarantini et al., 2019). Functional magnetic resonance imaging (fMRI) studies have demonstrated that hemodynamic response function (HRF) related parameters undergo age-related changes, with older adults exhibiting longer time-to-peak and lower peak amplitude compared to young adults (West et al., 2019; Stefanova et al., 2013). Additionally, transcranial Doppler (TCD) studies have revealed that older adults show reduced cerebral blood flow and slower blood flow velocity (Topcuoglu et al., 2009; Flück et al., 2014). Although increasing evidence has uncovered age-related NVC changes, these approaches primarily rely on indirect assessment through cerebrovascular parameters, while studies simultaneously recording neural electrical activity and hemodynamic changes are still limited.

Entropy is commonly employed in signal analysis as a measure of information contained in a signal, quantifying the predictability of future values based on the probability distribution of values already observed (Cacciotti et al., 2024). Given the nonlinear chaotic characteristics of neural systems, applying nonlinear entropy analysis to brain activity research facilitates understanding the complexity associated with neuronal activity variations (Bein, 2006). Some studies have utilized entropy analysis to reveal age-related complexity changes in Electroencephalogram (EEG) signals. Alù et al. (Alù et al., 2021) found that older adults exhibit higher approximate entropy in central, parietal, and occipital regions compared to young adults. Building upon this, Pappalettera et al. (Pappalettera et al., 2023) further demonstrated significant entropy across different EEG frequency bands. Beyond quantifying EEG signal complexity, entropy can also assess mutual influences between signals. Perpetuini et al. (Perpetuini et al., 2020) applied entropy to analyze EEG and functional near-infrared spectroscopy (fNIRS) signal complexity, revealing NVC changes in Alzheimer’s disease (AD) patients. Although entropy analysis has been widely adopted for EEG complexity studies, its application in NVC research remains limited. Only a few studies on neurological disorders and anesthesia have simultaneously acquired EEG and fNIRS signals, reporting NVC changes under these pathological and anesthetic conditions (Perpetuini et al., 2020; Liang et al., 2023; Vijayakrishnan Nair et al., 2021). However, investigations into healthy aging-related NVC changes remain insufficient.

This study aims to investigate age-related changes in NVC during healthy aging. We hypothesize that the complexity of EEG and fNIRS signals changes with aging, and that NVC would be negatively affected by aging. To test these hypotheses, we employed a multimodal EEG-fNIRS approach to systematically examine differences in signal complexity and NVC between young and older adults during four cognitive tasks. This approach enabled quantitative assessment of brain activity complexity during aging, providing novel evidence for understanding age-related NVC changes.

## 2 Materials and methods

### 2.1 Participants

This study recruited a total of 40 participants, comprising 20 young adults and 20 older adults (see Table 1 for demographic information). The research was conducted at the Department of Neurology, Peking Union Medical College Hospital. All participants underwent screening for cognitive status and medical history to determine eligibility before being included. Inclusion criteria included the ability to read and write in Chinese, sufficient hearing and vision for examination requirements, and good limb motor function. Individuals with any of the following conditions were excluded: dementia, behavioral or mental disorders, brain injury, or history of stroke. This study was approved by the Ethics Committee of Peking Union Medical College Hospital and conducted in accordance with the principles of the Declaration of Helsinki. All participants provided written informed consent before the experiment and were allowed to withdraw at any time.

**Table 1:** Demographic information.

Characteristic	Young *n* = 20	Old *n* = 20	*p*-value
Age, y (mean ± SD)	31.800 ± 6.910	68.150 ± 3.746	<0.001
Male, *n* (%)	14(70)	7 (35)	0.027
Education, y (mean ± SD)	13.800 ± 1.704	8.600 ± 1.603	<0.001

### 2.2 Task design and regions of interest

All participants were seated in chairs positioned at a table with a computer screen placed 80 cm away and completed the following four tasks: (1) auditory attention task, (2) verbal fluency task, (3) upper limb motor task, and (4) recognition memory task. Before formal testing, we provided detailed explanations of procedures and requirements for each task, followed by practice sessions (see Figure 1). All tasks were designed and presented using E-Prime software (Psychology Software Tools, Inc., Pittsburgh, PA, USA).

1. Auditory Attention Task: The paradigm comprised 6 task blocks (26 s each) alternating with 20s silent rest intervals, plus 20s pre-task and post-task silent periods. Each block contained 12 auditory stimuli (9 bilateral 0.5 kHz low-frequency tones and 3 bilateral 1 kHz high-frequency tones) presented in pseudo-random order. Participants were instructed to press the left mouse button for low-frequency tones and the right button for high-frequency tones. The complete paradigm lasted approximately 5 min. Region of interest (ROI) of this task included the prefrontal cortex (Fritz et al., 2007).
2. Verbal Fluency Task: The paradigm consisted of 6 task blocks (15 s each) alternating with 15 s rest intervals, plus 20s pre-task and post-task rest periods. Task blocks included fruit, flower, vegetable, furniture, stationery, and animal. Blocks contained pseudo-random combinations of these categories, with participants required to generate valid words. The number of correct responses per block was recorded. The complete paradigm lasted approximately 4 min. ROIs of this task included the dorsolateral prefrontal cortex and middle temporal gyrus (Brickman et al., 2005; Arrigo et al., 2024).
3. Upper Limb Motor Task: The paradigm consisted of 8 task blocks (15 s each) alternating with 15 s rest intervals, plus 20s pre-task and post-task rest periods. Each block required unilateral fist-clenching (4 left-hand or 4 right-hand blocks) presented in pseudo-random order. The complete paradigm lasted approximately 5 min. ROIs of this task included the bilateral primary motor cortex, ventral premotor cortex, and supplementary motor area (Bönstrup et al., 2016).
4. Recognition Memory Task: The paradigm consisted of 3 memory encoding blocks (22 s each) and 3 recognition blocks (22 s each) alternating with 20s rest intervals, plus 20s pre-task and post-task rest periods. Each encoding block contained 10 trials of 2 s-duration emotionally neutral color object images, requiring participants to indicate their personal preference via the left mouse button (“like”) or right button (“dislike”). Each recognition block also comprised 10 trials of 2 s-duration emotionally neutral color object images, containing 5 repeated images from the encoding phase and 5 novel images, requiring participants to make old/new discriminations by pressing the left mouse button (“old”) or the right button (“new”). The complete paradigm lasted approximately 5 min. ROIs of this task included the prefrontal and posterior parietal cortices (Eichenbaum, 2017; Wagner et al., 2005).

**Figure 1:**
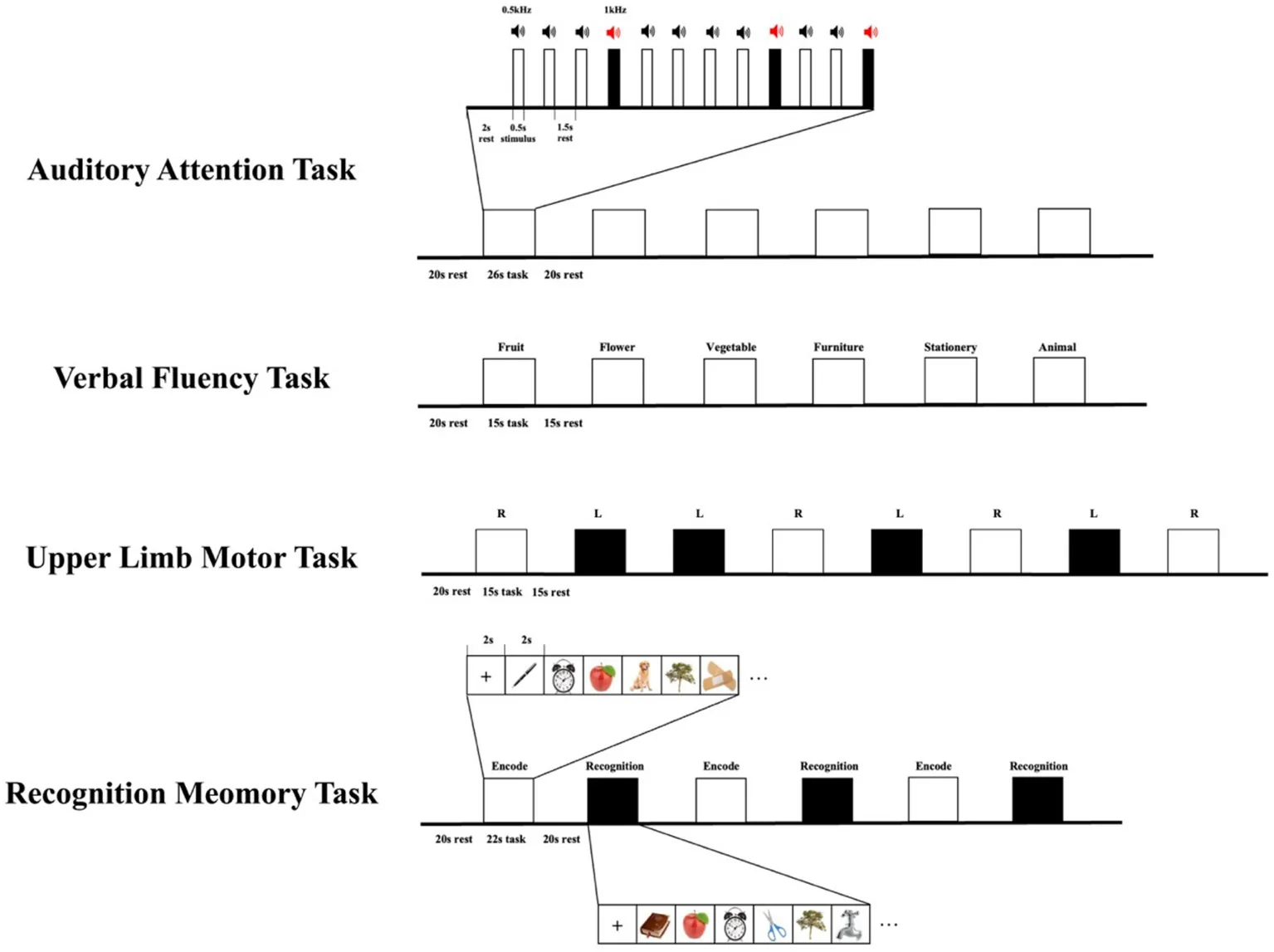
Task designs. This study employed four distinct task paradigms. The auditory attention task consisted of 26 s task blocks alternating with 20s rest intervals, with low-frequency (0.5 kHz) and high-frequency (1 kHz) tones presented in pseudo-random order within each block. The verbal fluency task involved 15 s task blocks alternating with 15 s rest intervals, with blocks presented in pseudo-random sequences. The upper limb motor task comprised 15 s task blocks alternating with 15 s rest intervals, containing pseudo-random ordered left-hand and right-hand fist-clenching trials. The recognition memory task consisted of 22 s encoding and recognition blocks, alternating with 20s rest intervals, during which emotionally neutral object images were displayed in pseudo-random sequences.

### 2.3 EEG and fNIRS instrumentation

The EEG data were acquired using a 64-channel Synamps2 system (Neuroscan, EI Paso, TX, USA) with electrodes arranged according to the international 10–10 system. The reference electrode was positioned at the midpoint between the Pz and CPz electrodes (see reference electrode diagram). Continuous recordings were obtained at a sampling rate of 1,000 Hz with scalp-electrode impedances maintained below 5 kΩ. All signals were acquired and monitored in real-time using Curry 8 software (see Figure 2).

**Figure 2:**
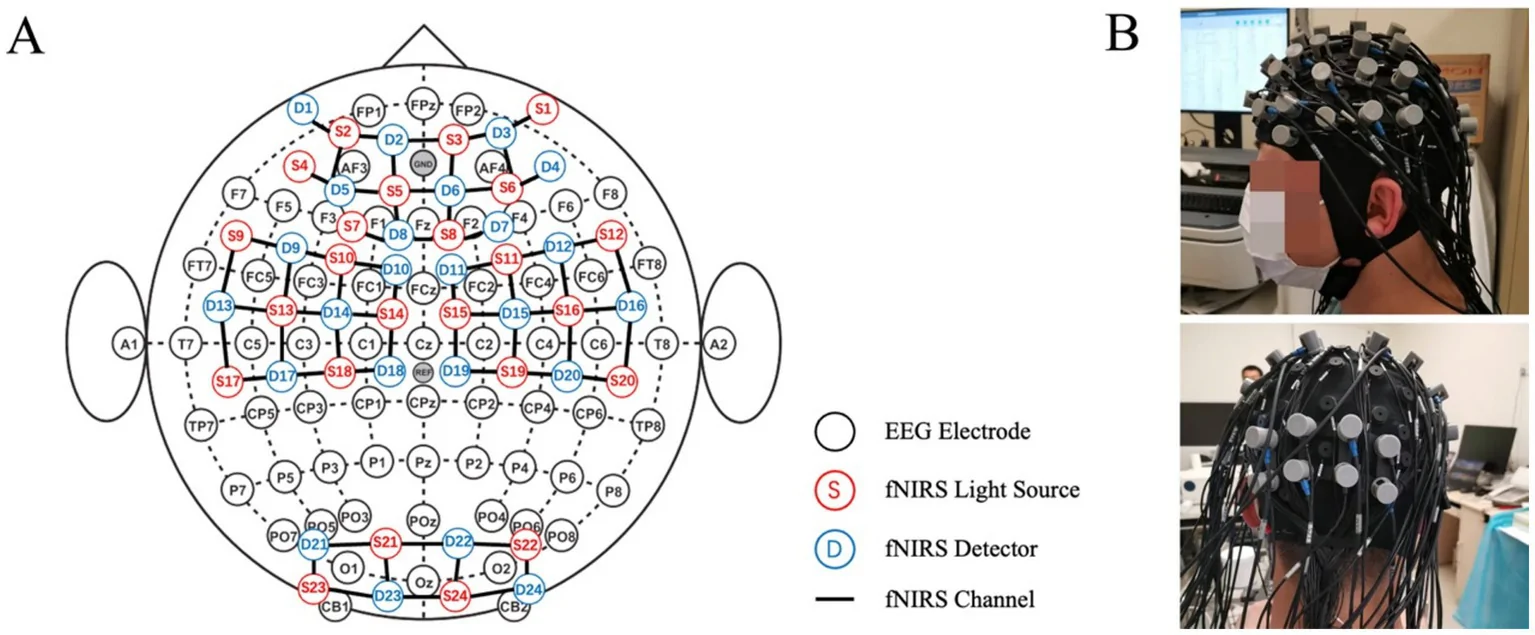
Acquisition device and distribution of EEG electrodes and fNIRS probes. **(A)** The EEG electrode distribution follows the 10–10 system, and the fNIRS probes were positioned with reference to the EEG electrodes. **(B)** Schematic diagram of the combined EEG and fNIRS signal acquisition device.

The fNIRS data were collected using a multichannel fNIRS system (NirSpark, Danyang Huichuang Medical Equipment Co., Ltd., China) comprising 24 light sources and 24 detectors forming 63 measurement channels. The optode array covered the bilateral prefrontal lobe, middle-inferior frontoparietal lobe, temporal lobe, and occipital lobe (Figure 2). The source-detector distance was 30 mm, with a sampling frequency of 11 Hz using dual wavelengths (730 nm and 850 nm). Relative concentration changes of oxygenated hemoglobin (HbO) and deoxygenated hemoglobin (HbR) were calculated online using the modified Beer–Lambert law and visualized in real-time through the proprietary NirSpark software (see Figure 2).

### 2.4 EEG signal pre-processing

First, the EEG data were downsampled to 200 Hz and band-pass filtered between 0.5 and 40 Hz. Poor-quality EEG segments were manually identified and removed, and bad channels were interpolated. The Automatic Artifact Removal (AAR) toolbox was then used to reduce ocular and muscle artifacts (Gomez-Herrero, 2007), while the CleanLine plugin was applied separately to suppress power-line noise.

### 2.5 fNIRS signal pre-processing

The raw fNIRS signals were converted to optical density (OD). Motion artifacts were then removed using a wavelet-based method, followed by bandpass filtering of the OD data with a zero-phase fourth-order Butterworth digital filter (cut-off frequencies of 0.01 Hz and 0.1 Hz). After filtering, poor-quality segments and channels were manually rejected. Oscillation in the concentration of HbO and HbR was calculated for each channel using the modified Beer–Lambert law.

### 2.6 Complexity analysis

All complexity analyses were performed using the EntropyHub toolbox in MATLAB (Flood and Grimm, 2021). Sample entropy (SampEn) and conditional entropy (CondEn) are metrics for quantifying the complexity of individual time series. Cross-sample entropy (Cross-SampEn) and cross-conditional entropy (Cross-CondEn) are complexity measures that assess the coupling strength between two distinct time series (Richman and Moorman, 2000; Porta et al., 1999; Porta et al., 1998).

SampEn is defined as the negative natural logarithm of the conditional probability that two template vectors of length m matching within a tolerance r will continue to match when their length increases to m + 1 (Richman and Moorman, 2000). For a time series of *N* points *u*(*j*), SampEn is computed employing the following set of Equations 1–3:

$$\text{SampEn}(m,r,N)=-\frac{\ln A^{m}(r)}{B^{m}(r)}$$ (1)

$$A^{m}(r)={\left(N-m\right)}^{-1}\sum_{i=1}^{N-m}C_{i}^{m+1}(r)$$ (2)

$$B^{m}(r)={\left(N-m\right)}^{-1}\sum_{i=1}^{N-m}C_{i}^{m}(r)$$ (3)

Here, *N* denotes the total number of data points in the time series. *m* is the embedded dimension, which represents the number of consecutive data points used to construct a pattern. *r* is the tolerance factor, which is the threshold distance used to determine if two data points match. We defined $C_{i}^{m}(r)$ as (*N*−*m*−1)^−1^ times the number of vectors X_m_(*j*) within *r* of X_m_(*i*), where *j* ranges from 1 to *N*−*m*, and *j* ≠ *i* to exclude self-matches.

Cross-SampEn of two time series of N points *u*(*j*) and *v*(*j*) is computed employing the following set of Equations 4–6:

$$\text{Cross}-\text{SampEn}(m,r,N)(v\|u)=-\ln\frac{A^{m}(r)(v\|u)}{B^{m}(r)(v\|u)}$$ (4)

$$A^{m}(r)(v\|u)={\left(N-m\right)}^{-1}\sum_{i=1}^{N-m}C_{i}^{m+1}(r)(v\|u)$$ (5)

$$B^{m}(r)(v\|u)={\left(N-m\right)}^{-1}\sum_{i=1}^{N-m}C_{i}^{m}(r)(v\|u)$$ (6)

In defining cross-SampEn, we set $C_{i}^{m}(r)(\mathrm{vu})$ as ${\left(N-m\right)}^{-1}$ times the number of vectors *y_m_*(*j*) within *r* of *x_m_*(*i*), where *j* ranges from 1 to *N*−*m*.

CondEn quantifies the uncertainty of the L-th data point conditional on the preceding L-1 data points in a time series (Porta et al., 1999; Porta et al., 1998). Its estimation for a time series of *L* points *s*(*i*) is based on an estimation of the Shannon entropy in an *L*-dimensional phase space, and it can be computed with the following set of Equations 7,8:

CondEn(L)=SE(L)−SE(L−1) (7)

$$\mathrm{SE}(L)=-\sum p[s_{L}(i)]\log[p(s_{L}(i))]$$ (8)

Where $\left[p(s_{L}(i))\right]$ indicates the joint probability of finding a specific $s_{L}(i)$ in the hyperspace, and the sum is extended for all possible patterns of length *L*.

To correct the bias in Equation 7, Porta et al. defined the corrected conditional entropy given by Equation 9:

Corrected−CondEn(L)=CondEn(L)+CondEn(1)⋅perc(L) (9)

Where CondEn(1) equals the unidimensional Shannon Entropy of the signal *SE*(1), and perc(L) is the rate of single points in the L-dimensional space.

Building upon the corrected-CondEn, the cross-corrected-CondEn quantifies the degree of coupling between two time series of L points *s*(*i*) and *r*(*i*). The Cross-CondEn used in this study refers to the corrected-cross-CondEn. It is computed as follows (Equation 10):

$$\begin{array}{l} \text{Cross}-\text{Corrected}-\text{CondE}n_{s|r}(L)=\text{Cross}-\text{CondE}n_{s|r}(L)+\\ \text{CondE}n_{s}(1)\cdot \mathrm{per}c_{s|r}(L) \end{array}$$ (10)

Where s∣r indicates the mixture of the samples used to form the patterns $\left([s(i)],r_{L-1}(i)\right)$ .

Lower SampEn and CondEn indicate a time series with stronger regularity and reduced complexity. After preprocessing, EEG signals were separated into five frequency bands (delta: 1–4 Hz, theta: 4–8 Hz, alpha: 8–12 Hz, beta: 12–30 Hz, and gamma: 30–40 Hz) using second-order Butterworth bandpass filters with forward–backward zero-phase filtering. The same filter type and order were used for all frequency bands. For all entropy analyses, repeated trials or blocks belonging to the same task condition were temporally aligned and averaged before entropy calculation, and the resulting condition-specific averaged time series was used for subsequent analyses. Hilbert amplitude envelopes were averaged across trials or blocks before global field potential (GFP) calculation. HbO and HbR signals were averaged separately across channels within each task-specific ROI. A Phillips-Perron-based routine was used to assess stationarity and apply linear detrending when the unit-root null hypothesis was not rejected. SampEn used *m* = 2, *τ* = 1 sample, and *r* = 0.2 × the population-normalized SD of the signal. Corrected CondEn used *m* = 2, *τ* = 1 sample, and six equal-width quantization bins (*c* = 6) (Perpetuini et al., 2020; Richman and Moorman, 2000; Porta et al., 1998).

For cross-modal analysis, each EEG GFP series was convolved with a HRF and then resampled to 11 Hz to match fNIRS. The EEG and corresponding ROI-averaged HbO or HbR series were matched in length, processed using the same stationarity routine, and z-standardized before cross-entropy estimation. Cross-SampEn used *m* = 2, *τ* = 1 sample, and the function’s default tolerance of 0.2 times its internally calculated pooled dispersion. Corrected Cross-CondEn used *m* = 2, *τ* = 1 sample, and *c* = 6. Lower Cross-SampEn and Cross-CondEn values indicate greater regularity and statistical dependence between the processed EEG and hemodynamic signals (Richman and Moorman, 2000; Porta et al., 1999; Porta et al., 1998).

### 2.7 Statistical analysis

Statistical analysis was performed using MATLAB. Demographic differences between the two groups were assessed using independent-samples *t*-tests for continuous variables and Pearson’s chi-square tests for categorical variables. Group differences in EEG, fNIRS, and cross-modal entropy metrics were examined using analysis of covariance (ANCOVA), with age group as the fixed factor, sex as a categorical variable, and years of education as a continuous covariate. Sex and years of education were included based on their potential influences on neural and hemodynamic activity. Previous studies have reported sex-related differences in neural oscillations and cerebrovascular physiology, whereas educational attainment is commonly used as a proxy for cognitive reserve and may be associated with task-related brain activity (Jausovec and Jausovec, 2010; Heinzel et al., 2013; Stern et al., 2020). The homogeneity of regression slopes assumption was assessed using the Group × Education interaction. Residual normality and homogeneity of variance were assessed using the Shapiro–Wilk test and median-centered Levene test, respectively. *p*-values for the group effects were adjusted for multiple comparisons using false discovery rate (FDR) correction. FDR-adjusted *p* values < 0.05 were considered statistically significant.

## 3 Results

### 3.1 Study participants

The demographic characteristics of participants are presented in Table 1. This research included 20 young (31.800 ± 6.910 years) and 20 older adults (68.150 ± 3.746 years). Significant differences were observed between groups in both age (*t* = −20.128, *p* < 0.001) and gender distribution (*χ*^2^ = 4.912, *p* = 0.027). Years of education were significantly higher in the young group than in the older group (13.800 ± 1.704 vs. 8.600 ± 1.603 years, t = 9.940, *p* < 0.001).

### 3.2 SampEn and CondEn of EEG

We first compared EEG SampEn and CondEn between young and older adults across the four cognitive tasks using ANCOVA with sex and years of education as covariates. Significant group differences were observed in the auditory attention task and the recognition memory task (see Figure 3).

**Figure 3:**
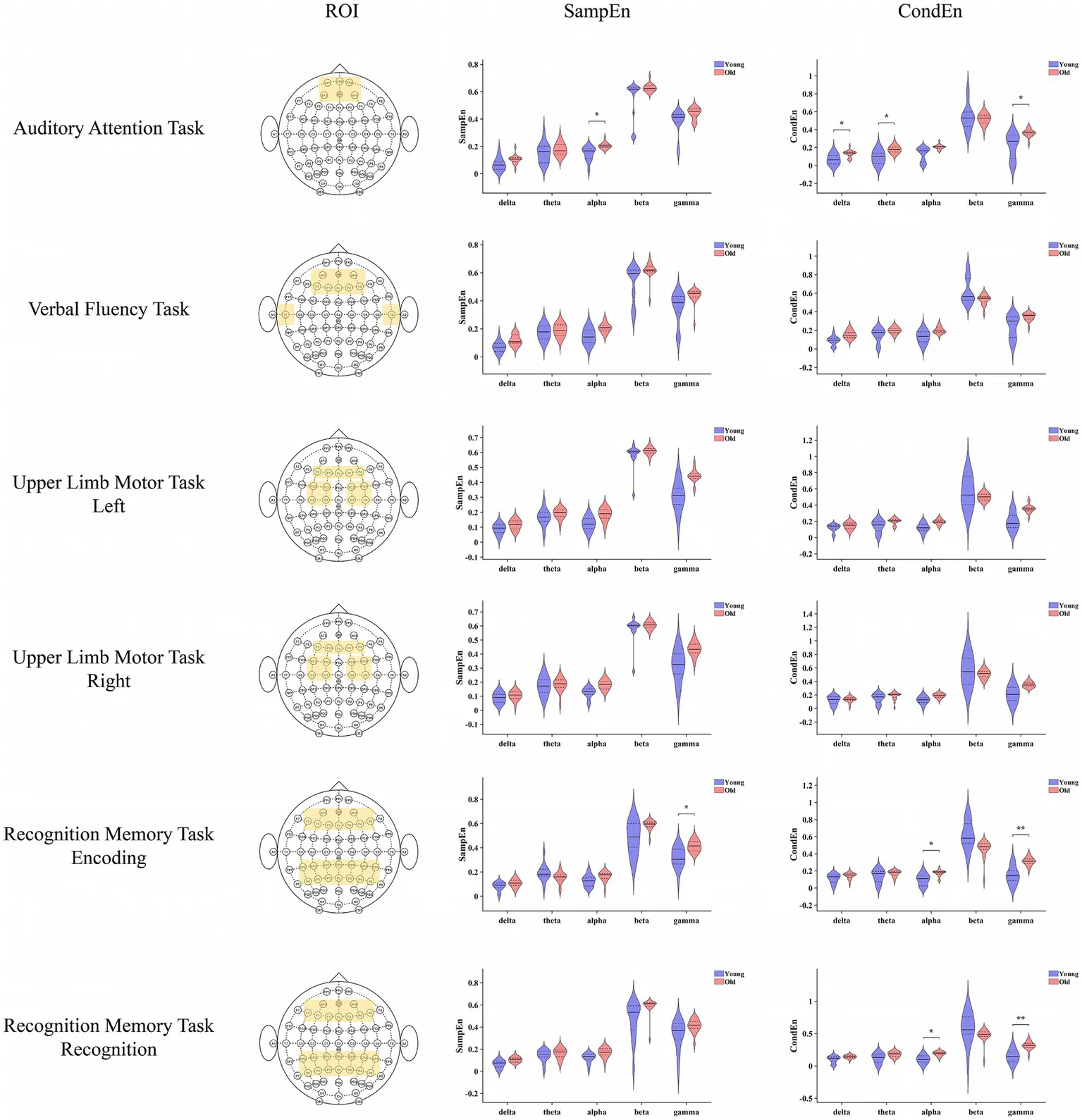
Results of EEG SampEn and CondEn. The yellow area represents the ROI corresponding to the task and its associated electrode positions. In the violin plots, the blue violins represent the SampEn and CondEn values of young adults, whereas the red violins represent those of older adults. The solid black line inside each violin denotes the median value, and the dashed black lines indicate the 25th and 75th percentiles of each group’s value distribution, respectively. *p < 0.05, **p < 0.01.

In the auditory attention task, older adults showed higher SampEn in the alpha band (B = 0.064, SE = 0.023, *F* = 7.842, *p* = 0.045, partial *η*^2^ = 0.213) and higher CondEn in the delta (B = 0.084, SE = 0.030, *F* = 7.658, *p* = 0.024, partial *η*^2^ = 0.209), theta (B = 0.105, SE = 0.035, *F* = 8.966, *p* = 0.024, partial *η*^2^ = 0.236), and gamma bands (B = 0.159, SE = 0.063, *F* = 6.441, *p* = 0.028, partial *η*^2^ = 0.182) compared with young adults. During the encoding phase of the recognition memory task, older adults showed higher SampEn in the gamma band (B = 0.114, SE = 0.039, *F* = 8.369, *p* = 0.035, partial *η*^2^ = 0.213), as well as higher CondEn in the alpha (B = 0.078, SE = 0.031, *F* = 6.153, *p* = 0.047, partial *η*^2^ = 0.166) and gamma bands (B = 0.177, SE = 0.046, *F* = 14.616, *p* = 0.003, partial *η*^2^ = 0.320).

During the recognition phase of the recognition memory task, older adults exhibited higher CondEn in the alpha (B = 0.087, SE = 0.029, *F* = 9.165, *p* = 0.012, partial *η*^2^ = 0.228) and gamma bands (B = 0.187, SE = 0.045, *F* = 17.668, *p* = 0.001, partial *η*^2^ = 0.363). No FDR-significant differences in EEG SampEn or CondEn were found between the two groups in the verbal fluency task or either upper limb motor task. No additional EEG entropy differences remained significant. For the full dataset, see Supplementary material.

### 3.3 SampEn and CondEn of fNIRS

We subsequently compared fNIRS SampEn and CondEn between young and older adults across the four cognitive tasks. Only one significant group difference remained. During the recognition phase of the recognition memory task, older adults exhibited lower HbO SampEn than young adults (B = −0.040, SE = 0.017, *F* = 5.800, *p* = 0.044, partial *η*^2^ = 0.158). No FDR-significant differences in fNIRS SampEn or CondEn were observed in the auditory attention task, verbal fluency task, either upper limb motor task, or the encoding phase of the recognition memory task (see Figure 4). For the full dataset, see Supplementary material.

**Figure 4:**
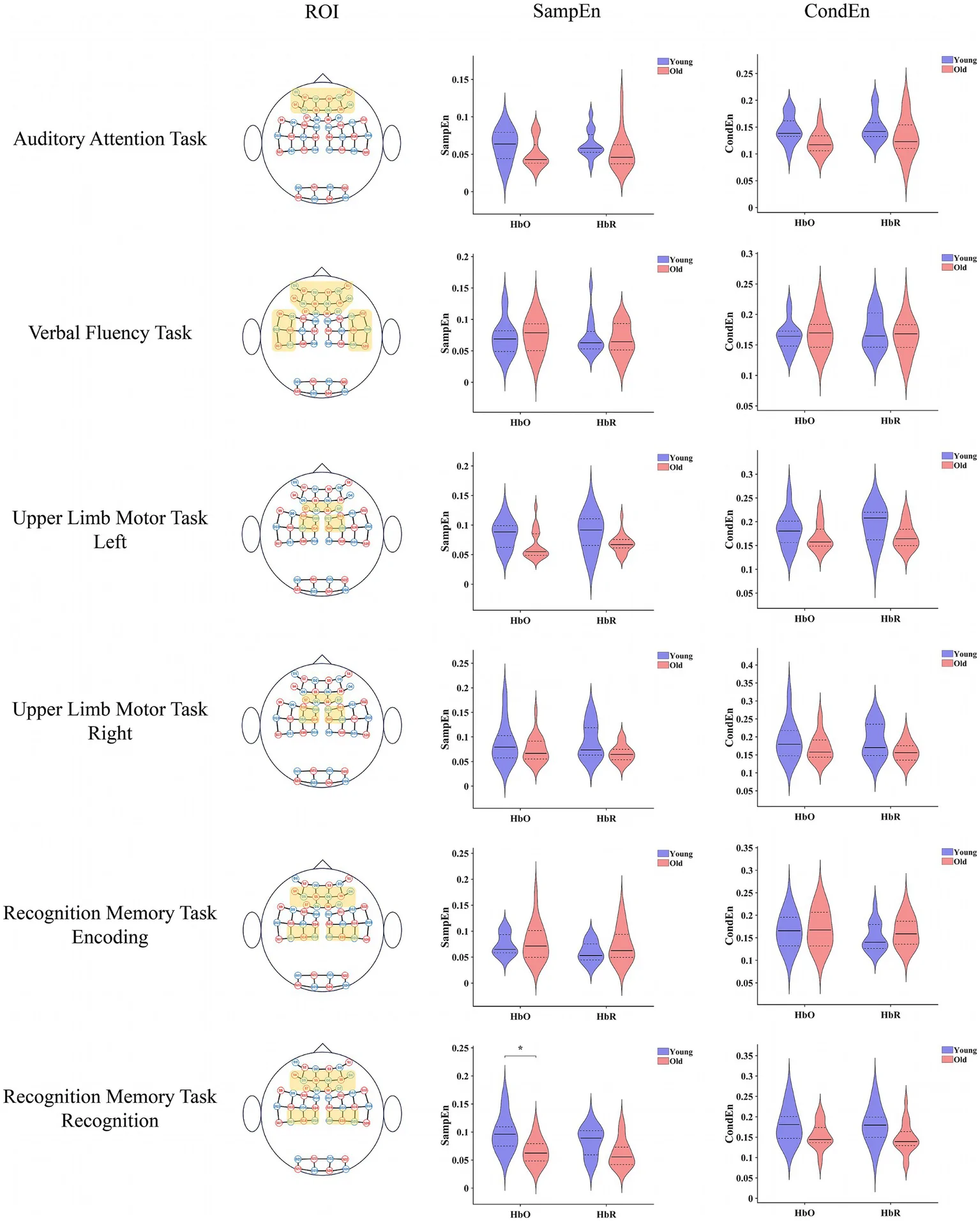
Results of fNIRS SampEn and CondEn. The yellow area represents the ROI corresponding to the task and its associated fNIRS channels. In the violin plots, the blue violins represent the SampEn and CondEn values of young adults, whereas the red violins represent those of older adults. The solid black line inside each violin denotes the median value, and the dashed black lines indicate the 25th and 75th percentiles of each group’s value distribution, respectively. *p < 0.05, **p < 0.01.

### 3.4 Cross-SampEn and cross-CondEn of EEG and fNIRS

We finally compared Cross-SampEn and Cross-CondEn between young and older adults across the four cognitive tasks. Significant group differences were confined to Cross-SampEn in the left upper limb motor task and the encoding phase of the recognition memory task (see Figure 5).

**Figure 5:**
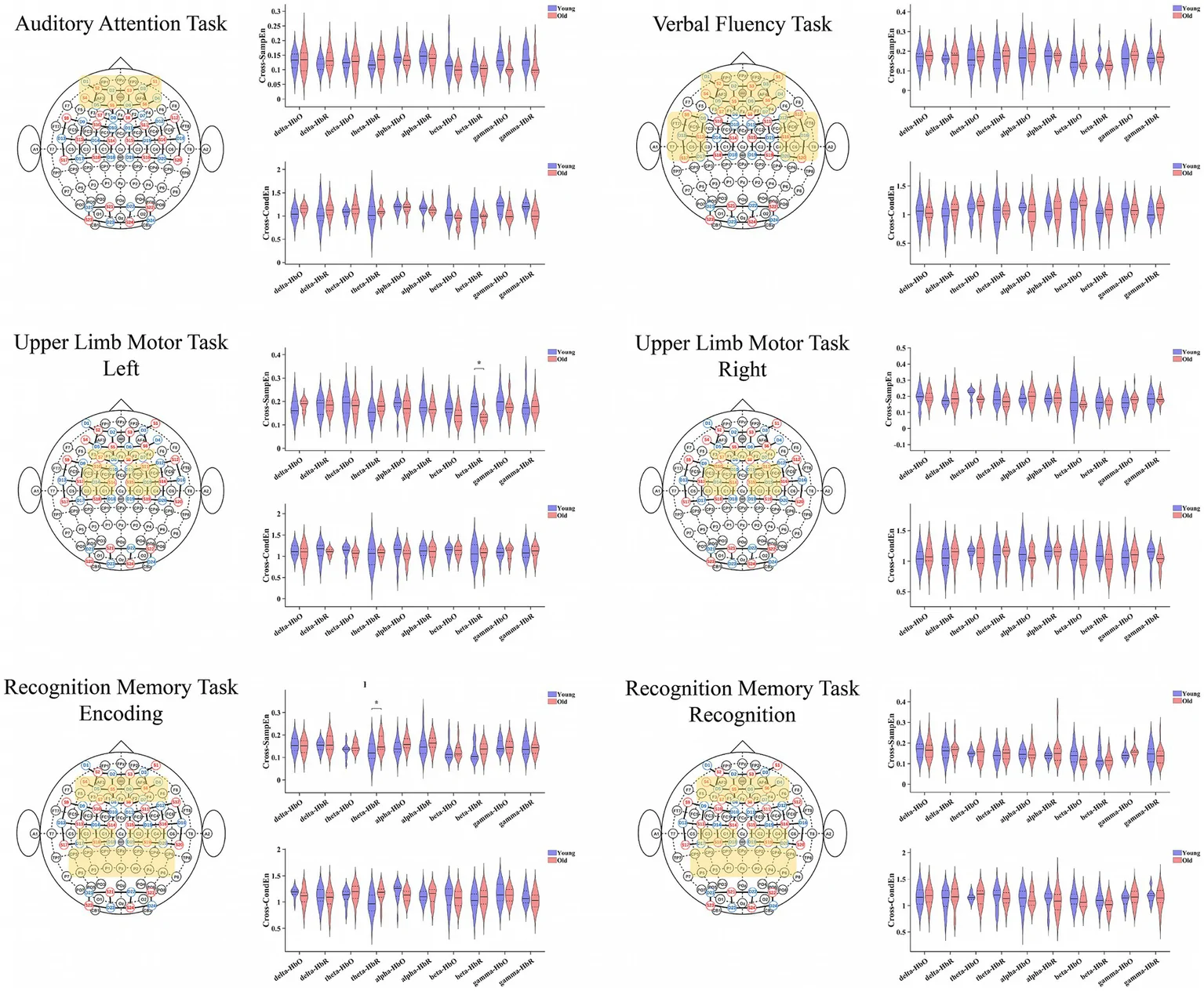
Results of Cross-SampEn and Cross-CondEn. The yellow area represents the ROI corresponding to the task and its associated EEG electrodes and fNIRS channels. In the violin plots, the blue violins represent the Cross-SampEn and Cross-CondEn values of young adults, whereas the red violins represent those of older adults. The solid black line inside each violin denotes the median value, and the dashed black lines indicate the 25th and 75th percentiles of each group’s value distribution, respectively. *p < 0.05, **p < 0.01.

In the left upper limb motor task, older adults showed lower Cross-SampEn between the beta band and HbR than young adults (B = −0.070, SE = 0.021, *F* = 11.051, *p* = 0.025, partial *η*^2^ = 0.283).

During the encoding phase of the recognition memory task, older adults showed higher Cross-SampEn between the theta band and HbR than young adults (B = 0.072, SE = 0.023, *F* = 9.933, *p* = 0.036, partial *η*^2^ = 0.243). No FDR-significant differences were observed during the recognition phase of the recognition memory task.

No FDR-significant group differences in either Cross-SampEn or Cross-CondEn were observed in the auditory attention task, verbal fluency task, or right upper limb motor task. In addition, no Cross-CondEn differences remained significant in any task or task phase (see Figure 5). For the 12 primary significant outcomes, no Group × Education interaction was statistically significant (see Supplementary Table S19). For the full dataset, see Supplementary material.

## 4 Discussion

This study was based on simultaneous EEG-fNIRS recordings and investigated changes in NVC during healthy aging from three levels: the complexity of neural electrical activity, the complexity of hemodynamic activity, and the cross-modal dynamic relationship between EEG and fNIRS. Age-related differences were mainly observed in the auditory attention task, motor task, and memory task. The EEG results showed that older adults exhibited higher SampEn or CondEn in some frequency bands during multiple task phases. The fNIRS results showed that older adults had lower HbO SampEn during the recognition phase of the recognition memory task. Cross-modal analysis showed lower beta-HbR Cross-SampEn in older adults during the left upper limb motor task and higher theta-HbR Cross-SampEn during memory encoding. These findings suggest that age-related changes in NVC are not manifested as a simple and uniform enhancement or reduction, but are more likely to reflect task-specific reorganization of neural activity, hemodynamic responses, and the dynamic matching between the two modalities.

At the EEG level, older adults showed higher entropy values for several significant measures, particularly during auditory attention and memory processing. Higher SampEn or CondEn indicates that the time series of neural electrical activity is more complex and less predictable, suggesting that the older brain may adopt more diverse patterns of neural activity during task states. Previous studies have shown that changes in EEG complexity during normal aging are clearly dependent on frequency band and task, with some studies reporting increased neural signal entropy or variability in older adults and others observing reduced complexity (Cacciotti et al., 2024; Alù et al., 2021; Pappalettera et al., 2023). Task-related changes in EEG complexity have also been reported in healthy young adults, with verbal cognitive load producing a reduction in fractal complexity compared with the resting state, further indicating that brain complexity is strongly dependent on the current cognitive state and task demands (Özel and Kazdağlı, 2026). This inconsistency indicates that aging does not cause neural activity complexity to change in a single direction, but is more likely to lead to readjustment of the organization of neural dynamics (McIntosh et al., 2014). In particular, in the memory task, the present study observed increased SampEn or CondEn in multiple frequency bands, suggesting that neural activity patterns in older adults are more dispersed and variable during memory encoding and recognition. Because neural activity is the upstream driver of local hemodynamic responses, such changes in neural dynamic patterns may further influence the temporal structure and stability of NVC.

The fNIRS results showed that older adults had lower HbO SampEn during the recognition phase of the recognition memory task. Lower HbO SampEn indicates that the hemodynamic response is more regular and that its temporal complexity is reduced. Effective neurovascular coupling depends on the dynamic matching among neural activity, local blood flow, and oxygen metabolism. Therefore, reduced hemodynamic complexity may imply a narrowing of the variability and dynamic regulatory range of vascular response (Mukli et al., 2024; Zimmerman et al., 2021). Previous studies have shown that healthy aging can be accompanied by changes in the amplitude and temporal characteristics of hemodynamic responses and in the regulation of cerebral blood flow (West et al., 2019; Stefanova et al., 2013; Beishon et al., 2022). Therefore, the reduced HbO SampEn during memory recognition observed in this study may reflect a tendency toward more fixed hemodynamic responses in older adults under cognitive load, thereby altering the temporal flexibility of the NVC system.

Notably, the directions of change in EEG and fNIRS entropy were not consistent. Some EEG entropy measures were increased in older adults, whereas HbO entropy during memory recognition was reduced. This difference suggests that neural activity and hemodynamic activity do not necessarily change synchronously during aging. Neural activity may exhibit higher temporal complexity and greater dynamic variability, whereas the hemodynamic response tends to become more regular. It is precisely this inconsistency in the direction of changes between the two modalities that suggests that the matching relationship between neural activity and hemodynamic responses may be reorganized during aging. Therefore, analysis of EEG or fNIRS alone may be insufficient to fully describe age-related changes in NVC, whereas cross-modal analysis can further reveal alterations in the dynamic relationship between the two.

The cross-modal results further support this point. During the left upper limb motor task, older adults showed lower beta-HbR Cross-SampEn. Lower Cross-SampEn indicates greater regularity and statistical dependence between the processed EEG and HbR time series, meaning that a more stable and predictable temporal relationship is formed between beta-related neural activity and the hemodynamic response. Beta rhythms are closely related to motor preparation, motor execution, and maintenance of sensorimotor system states (Engel and Fries, 2010; Kilavik et al., 2013). Therefore, this finding suggests that, with increasing age, the dynamic matching pattern between motor-related neural activity and hemodynamic responses may become more fixed. This phenomenon can be related to the concept of age-related physiological system “decomplexification,” in which interaction patterns among multiple systems gradually become more regular and stereotyped and the degrees of dynamic freedom decrease (Lipsitz and Goldberger, 1992; Goldberger et al., 2002). From the perspective of NVC, this change is more likely to reflect reorganization of the motor-related neurovascular response rather than simply stronger coupling.

In contrast, older adults showed higher theta-HbR Cross-SampEn during memory encoding, indicating that the joint dynamics between theta-related neural activity and the HbR response were more complex and that their temporal correspondence was more variable. Theta activity plays an important role in episodic memory encoding, information organization, and hippocampal-cortical interactions (Eichenbaum, 2017; Klimesch, 1999; Herweg et al., 2020). Therefore, this finding may indicate that, with increasing age, the matching relationship between neural activity and hemodynamic responses during memory encoding becomes more distributed. Unlike the relatively regularized cross-modal relationship observed during the motor task, memory encoding involves more complex cognitive processing and coordination among multiple brain regions. Its NVC may therefore be more susceptible to the combined influence of age-related neural and vascular changes, resulting in greater cross-modal complexity.

The opposite directions of Cross-SampEn changes during the left upper limb motor task and memory encoding represent one of the meaningful findings of this study. This indicates that age-related changes in NVC are clearly task dependent and do not follow a uniform pattern of “enhancement” or “reduction.” In the motor system, the relationship between neural and hemodynamic activity may become more regular and fixed, whereas during memory encoding the dynamic correspondence between the two may become more variable. This difference suggests that normal aging is more likely to lead to dynamic remodeling of NVC rather than unidirectional impairment. Previous studies have also shown that age-related changes in NVC are not completely consistent across different cognitive tasks, motor tasks, and brain regions (Sorond et al., 2008; Csipo et al., 2019). Therefore, conceptualizing age-related NVC as a reorganization process that depends on task demands and the characteristics of the neural system may be more consistent with the available evidence (Kannurpatti et al., 2010; Zaletel et al., 2005; Stefanidis et al., 2019; Grinband et al., 2017).

In addition, no significant group differences in Cross-CondEn remained, whereas Cross-SampEn still showed task-specific differences. This suggests that different cross-modal entropy measures may have different sensitivities to different aspects of NVC dynamics. Cross-SampEn focuses more on the overall regularity of joint patterns between two time series (Richman and Moorman, 2000), whereas Cross-CondEn places greater emphasis on uncertainty under conditional states (Porta et al., 1999; Porta et al., 1998). Therefore, the current results suggest that aging may have a more evident influence on the overall temporal structure between EEG and fNIRS than on condition-dependent structure. Future studies combining correlation analysis, time-lag analysis, general linear model, or other NVC assessment methods may help validate these cross-modal findings from different perspectives. Taken together, these findings further support the task-dependent nature of age-related changes in NVC.

This study still has several limitations. First, the sample size was relatively limited, and the young and older groups differed in sex distribution and years of education. Although analysis of covariance was used to adjust for sex and years of education, future studies are still needed to validate the stability of the current findings in larger cohorts with more balanced demographic characteristics. Second, task behavioral performance was not systematically associated with the entropy and cross-modal measures in the present study; therefore, the current analysis mainly describes age-related changes from the perspectives of neural electrical activity, hemodynamic activity, and their dynamic relationship. Future studies combining cognitive task performance and longitudinal follow-up may help further clarify the relationships between these complexity and NVC measures and changes in cognitive function. Third, short-separation measurements and concurrent systemic physiological recordings were not available in the present study. Therefore, the potential contribution of extracerebral and systemic physiological signals to the fNIRS measurements cannot be completely excluded. Finally, the present cross-modal analyses were based on a canonical HRF applied consistently across participants, providing a common framework for group comparisons. Because previous studies have suggested age-related and interindividual variability in hemodynamic response timing and shape (West et al., 2019; Stefanova et al., 2013), future studies incorporating alternative HRF parameterizations or individually estimated hemodynamic delays may further characterize age-related variability in neural–hemodynamic dynamics. Given the relatively limited sample size and the multimodal, multi-parameter nature of the analyses, the present study should be considered exploratory.

## 5 Conclusion

In this study, simultaneous EEG-fNIRS recordings revealed task-specific alterations in neural, hemodynamic, and cross-modal dynamics associated with healthy aging. Older adults exhibited higher EEG SampEn or CondEn in selected frequency bands during auditory attention and memory processing, together with lower HbO SampEn during memory recognition, indicating increased complexity of neural activity in some task states but reduced temporal complexity of the hemodynamic response. Cross-modal analysis showed lower beta-HbR Cross-SampEn during the left upper limb motor task and higher theta-HbR Cross-SampEn during memory encoding, suggesting that aging affects the temporal relationship between neural and hemodynamic signals in a task-dependent and directionally heterogeneous manner. Overall, these findings suggest that age-related changes in NVC are better characterized as dynamic reorganization rather than a uniform enhancement or impairment, and demonstrate the potential of multimodal entropy analysis for characterizing age-related changes in neurovascular dynamics.

## Data Availability

The raw data supporting the conclusions of this article will be made available by the authors, without undue reservation.

## References

[ref1] Alù F. Orticoni A. Judica E. Cotelli M. Rossini P. M. Miraglia F. . (2021). Entropy modulation of electroencephalographic signals in physiological aging. Mech. Ageing Dev. 196:111472. doi: 10.1016/j.mad.2021.111472, 33766746

[ref2] Arrigo I. V. da Silva P. H. R. Leoni R. F. (2024). Functional and effective connectivity underlying semantic verbal fluency. Brain Topogr. 37, 1043–1054. doi: 10.1007/s10548-024-01059-x, 38839695

[ref3] Bein B. (2006). Entropy. Best Pract. Res. Clin. Anaesthesiol. 20, 101–109. doi: 10.1016/j.bpa.2005.07.009, 16634417

[ref4] Beishon L. C. Boadi E. Williams C. A. Chithiramohan T. Barnes S. C. Intharakham K. . (2022). Age-related differences in cerebrovascular responses to cognitive stimulation using a novel method. Neuropsychol. Dev. Cogn. B Aging Neuropsychol. Cogn. 29, 929–942. doi: 10.1080/13825585.2021.1934387, 34098843

[ref5] Bönstrup M. Schulz R. Feldheim J. Hummel F. C. Gerloff C. (2016). Dynamic causal modelling of EEG and fMRI to characterize network architectures in a simple motor task. NeuroImage 124, 498–508. doi: 10.1016/j.neuroimage.2015.08.052, 26334836

[ref6] Brickman A. M. Paul R. H. Cohen R. A. Brickman A. Paul R. Cohen R. . (2005). Category and letter verbal fluency across the adult lifespan: relationship to EEG theta power. Arch. Clin. Neuropsychol. 20, 561–573. doi: 10.1016/j.acn.2004.12.006, 15939182 PMC2758771

[ref7] Cacciotti A. Pappalettera C. Miraglia F. Rossini P. M. Vecchio F. (2024). EEG entropy insights in the context of physiological aging and Alzheimer’s and Parkinson’s diseases: a comprehensive review. Geroscience. 46, 5537–5557. doi: 10.1007/s11357-024-01185-1, 38776044 PMC11493957

[ref8] Csipo T. Mukli P. Lipecz A. Tarantini S. Bahadli D. Abdulhussein O. . (2019). Assessment of age-related decline of neurovascular coupling responses by functional near-infrared spectroscopy (fNIRS) in humans. Geroscience. 41, 495–509. doi: 10.1007/s11357-019-00122-x, 31676966 PMC6885078

[ref9] Dion-Albert L. Dudek K. A. Russo S. J. Campbell M. Menard C. (2023). Neurovascular adaptations modulating cognition, mood, and stress responses. Trends Neurosci. 46, 276–292. doi: 10.1016/j.tins.2023.01.005, 36805768

[ref10] Eichenbaum H. (2017). Memory: organization and control. Annu. Rev. Psychol. 68, 19–45. doi: 10.1146/annurev-psych-010416-044131, 27687117 PMC5499681

[ref11] Engel A. K. Fries P. (2010). Beta-band oscillations—Signalling the status quo? Curr. Opin. Neurobiol. 20, 156–165. doi: 10.1016/j.conb.2010.02.015, 20359884

[ref12] Flood M. W. Grimm B. (2021). EntropyHub: an open-source toolkit for entropic time series analysis. PLoS One 16:e0259448. doi: 10.1371/journal.pone.0259448, 34735497 PMC8568273

[ref13] Flück D. Beaudin A. E. Steinback C. D. Kumarpillai G. Shobha N. McCreary C. R. . (2014). Effects of aging on the association between cerebrovascular responses to visual stimulation, hypercapnia and arterial stiffness. Front. Physiol. 5:49. doi: 10.3389/fphys.2014.00049, 24600398 PMC3928624

[ref14] Fritz J. B. Elhilali M. David S. V. Shamma S. A. (2007). Auditory attention—focusing the searchlight on sound. Curr. Opin. Neurobiol. 17, 437–455. doi: 10.1016/j.conb.2007.07.011, 17714933

[ref15] Goldberger A. L. Peng C. K. Lipsitz L. A. (2002). What is physiologic complexity and how does it change with aging and disease? Neurobiol. Aging 23, 23–26. doi: 10.1016/s0197-4580(01)00266-4, 11755014

[ref16] Gomez-Herrero G. (2007). Automatic artifact removal (AAR) toolbox v1.3 (release 09.12.2007) for MATLAB. Tampere University of Technology. 3, 1–23.

[ref17] Grinband J. Steffener J. Razlighi Q. R. Stern Y. (2017). BOLD neurovascular coupling does not change significantly with normal aging. Hum. Brain Mapp. 38, 3538–3551. doi: 10.1002/hbm.23608, 28419680 PMC5882590

[ref18] Heinzel S. Metzger F. G. Ehlis A. C. Korell R. Alboji A. Haeussinger F. B. . (2013). Aging-related cortical reorganization of verbal fluency processing: a functional near-infrared spectroscopy study. Neurobiol. Aging 34, 439–450. doi: 10.1016/j.neurobiolaging.2012.05.021, 22770542

[ref19] Herweg N. A. Solomon E. A. Kahana M. J. (2020). Theta oscillations in human memory. Trends Cogn. Sci. 24, 208–227. doi: 10.1016/j.tics.2019.12.006, 32029359 PMC8310425

[ref20] Isaev N. K. Stelmashook E. V. Genrikhs E. E. (2019). Neurogenesis and brain aging. Rev. Neurosci. 30, 573–580. doi: 10.1515/revneuro-2018-0084, 30763272

[ref21] Jausovec N. Jausovec K. (2010). Resting brain activity: differences between genders. Neuropsychologia 48, 3918–3925. doi: 10.1016/j.neuropsychologia.2010.09.020, 20875436

[ref22] Kannurpatti S. S. Motes M. A. Rypma B. Biswal B. B. (2010). Neural and vascular variability and the fMRI-BOLD response in normal aging. Magn. Reson. Imaging 28, 466–476. doi: 10.1016/j.mri.2009.12.007, 20117893 PMC2860003

[ref23] Kilavik B. E. Zaepffel M. Brovelli A. MacKay W. A. Riehle A. (2013). The ups and downs of *β* oscillations in sensorimotor cortex. Exp. Neurol. 245, 15–26. doi: 10.1016/j.expneurol.2012.09.014, 23022918

[ref24] Klimesch W. (1999). EEG alpha and theta oscillations reflect cognitive and memory performance: a review and analysis. Brain Res. Brain Res. Rev. 29, 169–195. doi: 10.1016/S0165-0173(98)00056-3, 10209231

[ref25] Liang Z. Wang X. Yu Z. Tong Y. Li X. Ma Y. . (2023). Age-dependent neurovascular coupling characteristics in children and adults during general anesthesia. Biomed. Opt. Express 14, 2240–2259. doi: 10.1364/BOE.482127, 37206124 PMC10191645

[ref26] Lipsitz L. A. Goldberger A. L. (1992). Loss of ‘complexity’ and aging. Potential applications of fractals and chaos theory to senescence. JAMA 267, 1806–1809. doi: 10.1001/jama.1992.03480130122036, 1482430

[ref27] McIntosh A. R. Vakorin V. Kovacevic N. Wang H. Diaconescu A. Protzner A. B. (2014). Spatiotemporal dependency of age-related changes in brain signal variability. Cereb. Cortex 24, 1806–1817. doi: 10.1093/cercor/bht030, 23395850 PMC4051893

[ref28] Mukli P. Pinto C. B. Owens C. D. Csipo T. Lipecz A. Szarvas Z. . (2024). Impaired neurovascular coupling and increased functional connectivity in the frontal cortex predict age-related cognitive dysfunction. Adv Sci (Weinh). 11:e2303516. doi: 10.1002/advs.202303516, 38155460 PMC10962492

[ref29] Owens C. D. Pinto C. B. Mukli P. Gulej R. Velez F. S. Detwiler S. . (2024). Neurovascular coupling, functional connectivity, and cerebrovascular endothelial extracellular vesicles as biomarkers of mild cognitive impairment. Alzheimers Dement. 20, 5590–5606. doi: 10.1002/alz.14072, 38958537 PMC11350141

[ref30] Özel H. F. Kazdağlı H. (2026). Resting state is not actually a state of rest, as confirmed by the loss of physiological complexity in brain dynamics. Interdiscip. Med. J. 17, 10–18. doi: 10.17944/interdiscip.1676000

[ref31] Pappalettera C. Cacciotti A. Nucci L. Miraglia F. Rossini P. M. Vecchio F. (2023). Approximate entropy analysis across electroencephalographic rhythmic frequency bands during physiological aging of human brain. Geroscience. 45, 1131–1145. doi: 10.1007/s11357-022-00710-4, 36538178 PMC9886767

[ref32] Perpetuini D. Chiarelli A. M. Filippini C. Cardone D. Croce P. Rotunno L. . (2020). Working memory decline in Alzheimer’s disease is detected by complexity analysis of multimodal EEG-fNIRS. Entropy (Basel) 22:1380. doi: 10.3390/e22121380, 33279924 PMC7762102

[ref33] Porta A. Baselli G. Liberati D. Montano N. Cogliati C. Gnecchi-Ruscone T. . (1998). Measuring regularity by means of a corrected conditional entropy in sympathetic outflow. Biol. Cybern. 78, 71–78. doi: 10.1007/s004220050414, 9485587

[ref34] Porta A. Baselli G. Lombardi F. Montano N. Malliani A. Cerutti S. (1999). Conditional entropy approach for the evaluation of the coupling strength. Biol. Cybern. 81, 119–129. doi: 10.1007/s004220050549, 10481240

[ref35] Richman J. S. Moorman J. R. (2000). Physiological time-series analysis using approximate entropy and sample entropy. Am. J. Physiol. Heart Circ. Physiol. 278, H2039–H2049. doi: 10.1152/ajpheart.2000.278.6.H2039, 10843903

[ref36] Sorond F. A. Schnyer D. M. Serrador J. M. Milberg W. P. Lipsitz L. A. (2008). Cerebral blood flow regulation during cognitive tasks: effects of healthy aging. Cortex 44, 179–184. doi: 10.1016/j.cortex.2006.01.003, 18387547 PMC2398722

[ref37] Stefanidis K. B. Askew C. D. Klein T. Lagopoulos J. Summers M. J. (2019). Healthy aging affects cerebrovascular reactivity and pressure-flow responses, but not neurovascular coupling: a cross-sectional study. PLoS One 14:e0217082. doi: 10.1371/journal.pone.0217082, 31095646 PMC6522028

[ref38] Stefanova I. Stephan T. Becker-Bense S. Dera T. Brandt T. Dieterich M. (2013). Age-related changes of blood-oxygen-level-dependent signal dynamics during optokinetic stimulation. Neurobiol. Aging 34, 2277–2286. doi: 10.1016/j.neurobiolaging.2013.03.031, 23628145

[ref39] Stern Y. Arenaza-Urquijo E. M. Bartrés-Faz D. Belleville S. Cantilon M. Chetelat G. . (2020). Whitepaper: defining and investigating cognitive reserve, brain reserve, and brain maintenance. Alzheimers Dement. 16, 1305–1311. doi: 10.1016/j.jalz.2018.07.219, 30222945 PMC6417987

[ref40] Tarantini S. Valcarcel-Ares M. N. Toth P. Yabluchanskiy A. Tucsek Z. Kiss T. . (2019). Nicotinamide mononucleotide (NMN) supplementation rescues cerebromicrovascular endothelial function and neurovascular coupling responses and improves cognitive function in aged mice. Redox Biol. 24:101192. doi: 10.1016/j.redox.2019.101192, 31015147 PMC6477631

[ref41] Topcuoglu M. A. Aydin H. Saka E. (2009). Occipital cortex activation studied with simultaneous recordings of functional transcranial Doppler ultrasound (fTCD) and visual evoked potential (VEP) in cognitively normal human subjects: effect of healthy aging. Neurosci. Lett. 452, 17–22. doi: 10.1016/j.neulet.2009.01.030, 19444940

[ref42] Vijayakrishnan Nair V. Kish B. R. Yang H. S. Yang H.-C. Yu Z. Guo H. . (2021). Monitoring anesthesia using simultaneous functional near infrared spectroscopy and electroencephalography. Clin. Neurophysiol. 132, 1636–1646. doi: 10.1016/j.clinph.2021.03.025, 34034088

[ref43] Wagner A. D. Shannon B. J. Kahn I. Buckner R. L. (2005). Parietal lobe contributions to episodic memory retrieval. Trends Cogn. Sci. 9, 445–453. doi: 10.1016/j.tics.2005.07.001, 16054861

[ref44] West K. L. Zuppichini M. D. Turner M. P. Sivakolundu D. K. Zhao Y. Abdelkarim D. . (2019). BOLD hemodynamic response function changes significantly with healthy aging. NeuroImage 188, 198–207. doi: 10.1016/j.neuroimage.2018.12.012, 30529628 PMC6450381

[ref45] Zaletel M. Strucl M. Pretnar-Oblak J. Zvan B. (2005). Age-related changes in the relationship between visual evoked potentials and visually evoked cerebral blood flow velocity response. Funct. Neurol. 20, 115–120. 16324234

[ref46] Zimmerman B. Rypma B. Gratton G. Fabiani M. (2021). Age-related changes in cerebrovascular health and their effects on neural function and cognition: a comprehensive review. Psychophysiology 58:e13796. doi: 10.1111/psyp.13796, 33728712 PMC8244108

