# Supplementary material for: Multimodal EEG-fNIRS complexity analysis can reveal the neurovascular coupling change with aging
Source: Front Aging Neurosci. 2026 Oct 26;18:1912923. doi: 10.3389/fnagi.2026.1912923 (PMC13745489; doi:10.3389/fnagi.2026.1912923)
Supplement: Supplementary file 1 [file Data_Sheet_1.docx]

**The EEG electrodes and fNIRS probes corresponding to ROIs for different cognitive tasks**

1. **Auditory Attention Task**

EEG electrodes: FP1 FPZ FP2 AF3 AF4

fNIRS channels: 1 2 3 4 5 6 7 8 9 10 11 13 14 15

2. **Verbal Fluency Task**

EEG electrodes: AF3 AF4 F1 F2 F3 F4 T7 T8

fNIRS channels: 1 2 3 4 6 7 8 9 10 12 13 15 16 17 18 20 21 28 29 30 31 33 40 42 43 44 45 52 53

3. **Left Upper Limb Motor Task**

EEG electrodes: F1 F2 F3 F4 FC1 FC2 FC3 FC4 C1 C2 C3 C4

fNIRS channels: 12 16 17 18 23 24 25 27 34 35 37 38

4. **Right Upper Limb Motor Task**

EEG electrodes: F1 F2 F3 F4 FC1 FC2 FC3 FC4 C1 C2 C3 C4

fNIRS channels: 12 16 17 18 23 24 25 27 34 35 37 38

5. **Encoding Phase of Recognition Memory Task**

EEG electrodes: CP1 CP2 CP3 CP4 CP5 CP6 P1 P2 P3 P4 P5 P6 F1 F2 F3 F4 F5 F6 AF3 AF4

fNIRS channels: 4 8 10 13 14 15 16 18 33 36 39 43 46 47 48 49 50 51

6. **Recognition Phase of Recognition Memory Task**

EEG electrodes: CP1 CP2 CP3 CP4 CP5 CP6 P1 P2 P3 P4 P5 P6 F1 F2 F3 F4 F5 F6 AF3 AF4

fNIRS channels: 4 8 10 13 14 15 16 18 33 36 39 43 46 47 48 49 50 51

**Supplementary Figure 1 EEG electrodes and fNIRS channel numbers.**

**Supplementary Table 1 EEG Entropy Parameters in the Auditory Attention Task**

| **Task/Condition** | **Band** | **Hb** | **N young** | **N old** | **Young mean ± SD** | **Old mean ± SD** | **B (old vs. young)** | **95% CI** | **F** | **p** | **partial η²** | **FDR-adjusted p** |
| --- | --- | --- | --- | --- | --- | --- | --- | --- | --- | --- | --- | --- |
| **SampEn** | | | | | | | | | | | | |
| Auditory attention | Delta | — | 14 | 19 | 0.069 ± 0.046 | 0.105 ± 0.032 | 0.047 | [-0.005, 0.099] | 3.374 | 0.077 | 0.104 | 0.128 |
| Auditory attention | Theta | — | 14 | 19 | 0.151 ± 0.069 | 0.179 ± 0.045 | 0.028 | [-0.049, 0.104] | 0.540 | 0.469 | 0.018 | 0.469 |
| Auditory attention | Alpha | — | 14 | 19 | 0.148 ± 0.051 | 0.205 ± 0.022 | 0.064 | [0.017, 0.112] | 7.842 | 0.009 | 0.213 | **0.045** |
| Auditory attention | Beta | — | 14 | 19 | 0.534 ± 0.152 | 0.628 ± 0.029 | 0.076 | [-0.053, 0.205] | 1.448 | 0.239 | 0.048 | 0.298 |
| Auditory attention | Gamma | — | 14 | 19 | 0.381 ± 0.094 | 0.443 ± 0.042 | 0.090 | [-0.003, 0.184] | 3.940 | 0.057 | 0.120 | 0.128 |
| **CondEn** | | | | | | | | | | | | |
| Auditory attention | Delta | — | 14 | 19 | 0.069 ± 0.055 | 0.131 ± 0.039 | 0.084 | [0.022, 0.146] | 7.658 | 0.010 | 0.209 | **0.024** |
| Auditory attention | Theta | — | 14 | 19 | 0.091 ± 0.069 | 0.179 ± 0.040 | 0.105 | [0.033, 0.176] | 8.966 | 0.006 | 0.236 | **0.024** |
| Auditory attention | Alpha | — | 14 | 19 | 0.123 ± 0.079 | 0.206 ± 0.026 | 0.071 | [-0.002, 0.144] | 3.979 | 0.056 | 0.121 | 0.069 |
| Auditory attention | Beta | — | 14 | 19 | 0.537 ± 0.145 | 0.521 ± 0.057 | 0.004 | [-0.132, 0.139] | 0.003 | 0.956 | 0.000 | 0.956 |
| Auditory attention | Gamma | — | 14 | 19 | 0.215 ± 0.139 | 0.363 ± 0.043 | 0.159 | [0.031, 0.287] | 6.441 | 0.017 | 0.182 | **0.028** |

Note. Values are reported as mean ± SD. B is the adjusted difference between older and younger adults from ANCOVA with sex and years of education as covariates. The 95% CI refers to B. F, p and partial η² refer to the group effect. FDR-adjusted p values are reported; values <0.05 are shown in bold. —, not applicable.

**Supplementary Table 2 EEG Entropy Parameters in the Verbal Fluency Task**

| **Task/Condition** | **Band** | **Hb** | **N young** | **N old** | **Young mean ± SD** | **Old mean ± SD** | **B (old vs. young)** | **95% CI** | **F** | **p** | **partial η²** | **FDR-adjusted p** |
| --- | --- | --- | --- | --- | --- | --- | --- | --- | --- | --- | --- | --- |
| **SampEn** | | | | | | | | | | | | |
| Verbal fluency | Delta | — | 16 | 19 | 0.070 ± 0.037 | 0.120 ± 0.038 | 0.055 | [0.006, 0.105] | 5.260 | 0.029 | 0.145 | 0.144 |
| Verbal fluency | Theta | — | 16 | 19 | 0.174 ± 0.047 | 0.191 ± 0.041 | 0.016 | [-0.041, 0.073] | 0.339 | 0.564 | 0.011 | 0.564 |
| Verbal fluency | Alpha | — | 16 | 19 | 0.147 ± 0.053 | 0.206 ± 0.034 | 0.050 | [-0.005, 0.106] | 3.412 | 0.074 | 0.099 | 0.186 |
| Verbal fluency | Beta | — | 16 | 19 | 0.518 ± 0.138 | 0.619 ± 0.060 | 0.091 | [-0.043, 0.226] | 1.917 | 0.176 | 0.058 | 0.293 |
| Verbal fluency | Gamma | — | 16 | 19 | 0.351 ± 0.114 | 0.436 ± 0.054 | 0.068 | [-0.047, 0.184] | 1.456 | 0.237 | 0.045 | 0.296 |
| **CondEn** | | | | | | | | | | | | |
| Verbal fluency | Delta | — | 16 | 19 | 0.090 ± 0.047 | 0.155 ± 0.040 | 0.066 | [0.009, 0.123] | 5.661 | 0.024 | 0.154 | 0.079 |
| Verbal fluency | Theta | — | 16 | 19 | 0.149 ± 0.076 | 0.198 ± 0.035 | 0.054 | [-0.022, 0.129] | 2.104 | 0.157 | 0.064 | 0.157 |
| Verbal fluency | Alpha | — | 16 | 19 | 0.128 ± 0.066 | 0.200 ± 0.037 | 0.047 | [-0.016, 0.110] | 2.299 | 0.140 | 0.069 | 0.157 |
| Verbal fluency | Beta | — | 16 | 19 | 0.623 ± 0.142 | 0.533 ± 0.056 | -0.144 | [-0.281, -0.006] | 4.554 | 0.041 | 0.128 | 0.079 |
| Verbal fluency | Gamma | — | 16 | 19 | 0.238 ± 0.125 | 0.346 ± 0.042 | 0.118 | [0.001, 0.236] | 4.255 | 0.048 | 0.121 | 0.079 |

Note. Values are reported as mean ± SD. B is the adjusted difference between older and younger adults from ANCOVA with sex and years of education as covariates. The 95% CI refers to B. F, p and partial η² refer to the group effect. FDR-adjusted p values are reported; values <0.05 are shown in bold. —, not applicable.

**Supplementary Table 3 EEG Entropy Parameters in the Left Upper Limb Motor Task**

| **Task/Condition** | **Band** | **Hb** | **N young** | **N old** | **Young mean ± SD** | **Old mean ± SD** | **B (old vs. young)** | **95% CI** | **F** | **p** | **partial η²** | **FDR-adjusted p** |
| --- | --- | --- | --- | --- | --- | --- | --- | --- | --- | --- | --- | --- |
| **SampEn** | | | | | | | | | | | | |
| Left motor | Delta | — | 12 | 20 | 0.087 ± 0.033 | 0.116 ± 0.032 | 0.005 | [-0.037, 0.048] | 0.062 | 0.805 | 0.002 | 0.873 |
| Left motor | Theta | — | 12 | 20 | 0.166 ± 0.057 | 0.192 ± 0.033 | -0.005 | [-0.062, 0.053] | 0.026 | 0.873 | 0.001 | 0.873 |
| Left motor | Alpha | — | 12 | 20 | 0.123 ± 0.042 | 0.186 ± 0.042 | 0.022 | [-0.032, 0.076] | 0.680 | 0.416 | 0.024 | 0.694 |
| Left motor | Beta | — | 12 | 20 | 0.578 ± 0.087 | 0.610 ± 0.025 | -0.041 | [-0.109, 0.028] | 1.468 | 0.236 | 0.050 | 0.590 |
| Left motor | Gamma | — | 12 | 20 | 0.306 ± 0.098 | 0.442 ± 0.042 | 0.055 | [-0.030, 0.140] | 1.773 | 0.194 | 0.060 | 0.590 |
| **CondEn** | | | | | | | | | | | | |
| Left motor | Delta | — | 12 | 20 | 0.118 ± 0.047 | 0.146 ± 0.041 | -0.001 | [-0.060, 0.057] | 0.003 | 0.960 | 0.000 | 0.960 |
| Left motor | Theta | — | 12 | 20 | 0.139 ± 0.075 | 0.201 ± 0.040 | 0.026 | [-0.048, 0.100] | 0.529 | 0.473 | 0.019 | 0.789 |
| Left motor | Alpha | — | 12 | 20 | 0.121 ± 0.057 | 0.194 ± 0.032 | 0.023 | [-0.031, 0.077] | 0.782 | 0.384 | 0.027 | 0.789 |
| Left motor | Beta | — | 12 | 20 | 0.553 ± 0.180 | 0.501 ± 0.046 | -0.032 | [-0.190, 0.125] | 0.176 | 0.678 | 0.006 | 0.847 |
| Left motor | Gamma | — | 12 | 20 | 0.198 ± 0.109 | 0.355 ± 0.049 | 0.060 | [-0.034, 0.154] | 1.702 | 0.203 | 0.057 | 0.789 |

Note. Values are reported as mean ± SD. B is the adjusted difference between older and younger adults from ANCOVA with sex and years of education as covariates. The 95% CI refers to B. F, p and partial η² refer to the group effect. FDR-adjusted p values are reported; values <0.05 are shown in bold. —, not applicable.

**Supplementary Table 4 EEG Entropy Parameters in the Right Upper Limb Motor Task**

| **Task/Condition** | **Band** | **Hb** | **N young** | **N old** | **Young mean ± SD** | **Old mean ± SD** | **B (old vs. young)** | **95% CI** | **F** | **p** | **partial η²** | **FDR-adjusted p** |
| --- | --- | --- | --- | --- | --- | --- | --- | --- | --- | --- | --- | --- |
| **SampEn** | | | | | | | | | | | | |
| Right motor | Delta | — | 12 | 20 | 0.089 ± 0.037 | 0.105 ± 0.031 | 0.041 | [-0.003, 0.085] | 3.653 | 0.066 | 0.115 | 0.331 |
| Right motor | Theta | — | 12 | 20 | 0.174 ± 0.065 | 0.183 ± 0.047 | 0.039 | [-0.034, 0.112] | 1.172 | 0.288 | 0.040 | 0.426 |
| Right motor | Alpha | — | 12 | 20 | 0.125 ± 0.039 | 0.182 ± 0.029 | 0.020 | [-0.022, 0.062] | 0.940 | 0.341 | 0.032 | 0.426 |
| Right motor | Beta | — | 12 | 20 | 0.576 ± 0.098 | 0.605 ± 0.027 | -0.028 | [-0.109, 0.053] | 0.499 | 0.486 | 0.018 | 0.486 |
| Right motor | Gamma | — | 12 | 20 | 0.319 ± 0.114 | 0.436 ± 0.048 | 0.055 | [-0.048, 0.158] | 1.188 | 0.285 | 0.041 | 0.426 |
| **CondEn** | | | | | | | | | | | | |
| Right motor | Delta | — | 12 | 20 | 0.119 ± 0.063 | 0.134 ± 0.041 | 0.052 | [-0.014, 0.119] | 2.586 | 0.119 | 0.085 | 0.250 |
| Right motor | Theta | — | 12 | 20 | 0.150 ± 0.070 | 0.185 ± 0.058 | 0.071 | [-0.012, 0.154] | 3.035 | 0.092 | 0.098 | 0.250 |
| Right motor | Alpha | — | 12 | 20 | 0.126 ± 0.055 | 0.191 ± 0.032 | 0.040 | [-0.015, 0.096] | 2.189 | 0.150 | 0.073 | 0.250 |
| Right motor | Beta | — | 12 | 20 | 0.559 ± 0.199 | 0.508 ± 0.063 | -0.024 | [-0.202, 0.154] | 0.075 | 0.786 | 0.003 | 0.786 |
| Right motor | Gamma | — | 12 | 20 | 0.207 ± 0.120 | 0.351 ± 0.042 | 0.062 | [-0.040, 0.165] | 1.549 | 0.224 | 0.052 | 0.279 |

Note. Values are reported as mean ± SD. B is the adjusted difference between older and younger adults from ANCOVA with sex and years of education as covariates. The 95% CI refers to B. F, p and partial η² refer to the group effect. FDR-adjusted p values are reported; values <0.05 are shown in bold. —, not applicable.

**Supplementary Table 5 EEG Entropy Parameters in the Encoding Phase of the Recognition Memory Task**

| **Task/Condition** | **Band** | **Hb** | **N young** | **N old** | **Young mean ± SD** | **Old mean ± SD** | **B (old vs. young)** | **95% CI** | **F** | **p** | **partial η²** | **FDR-adjusted p** |
| --- | --- | --- | --- | --- | --- | --- | --- | --- | --- | --- | --- | --- |
| **SampEn** | | | | | | | | | | | | |
| Memory encoding | Delta | — | 15 | 20 | 0.082 ± 0.035 | 0.107 ± 0.032 | 0.028 | [-0.015, 0.070] | 1.767 | 0.194 | 0.054 | 0.323 |
| Memory encoding | Theta | — | 15 | 20 | 0.191 ± 0.069 | 0.153 ± 0.046 | -0.024 | [-0.098, 0.050] | 0.422 | 0.520 | 0.013 | 0.520 |
| Memory encoding | Alpha | — | 15 | 20 | 0.127 ± 0.047 | 0.165 ± 0.040 | 0.030 | [-0.026, 0.086] | 1.231 | 0.276 | 0.038 | 0.345 |
| Memory encoding | Beta | — | 15 | 20 | 0.482 ± 0.130 | 0.590 ± 0.040 | 0.079 | [-0.036, 0.193] | 1.969 | 0.170 | 0.060 | 0.323 |
| Memory encoding | Gamma | — | 15 | 20 | 0.321 ± 0.082 | 0.414 ± 0.043 | 0.114 | [0.034, 0.194] | 8.369 | 0.007 | 0.213 | **0.035** |
| **CondEn** | | | | | | | | | | | | |
| Memory encoding | Delta | — | 15 | 20 | 0.111 ± 0.054 | 0.145 ± 0.038 | 0.035 | [-0.023, 0.094] | 1.537 | 0.224 | 0.047 | 0.374 |
| Memory encoding | Theta | — | 15 | 20 | 0.138 ± 0.075 | 0.171 ± 0.036 | 0.030 | [-0.044, 0.103] | 0.679 | 0.416 | 0.021 | 0.465 |
| Memory encoding | Alpha | — | 15 | 20 | 0.098 ± 0.067 | 0.174 ± 0.037 | 0.078 | [0.014, 0.142] | 6.153 | 0.019 | 0.166 | **0.047** |
| Memory encoding | Beta | — | 15 | 20 | 0.621 ± 0.162 | 0.460 ± 0.102 | -0.057 | [-0.216, 0.101] | 0.548 | 0.465 | 0.017 | 0.465 |
| Memory encoding | Gamma | — | 15 | 20 | 0.149 ± 0.103 | 0.312 ± 0.048 | 0.177 | [0.083, 0.272] | 14.616 | <0.001 | 0.320 | **0.003** |

Note. Values are reported as mean ± SD. B is the adjusted difference between older and younger adults from ANCOVA with sex and years of education as covariates. The 95% CI refers to B. F, p and partial η² refer to the group effect. FDR-adjusted p values are reported; values <0.05 are shown in bold. —, not applicable.

**Supplementary Table 6 EEG Entropy Parameters in the Recognition Phase of the Recognition Memory Task**

| **Task/Condition** | **Band** | **Hb** | **N young** | **N old** | **Young mean ± SD** | **Old mean ± SD** | **B (old vs. young)** | **95% CI** | **F** | **p** | **partial η²** | **FDR-adjusted p** |
| --- | --- | --- | --- | --- | --- | --- | --- | --- | --- | --- | --- | --- |
| **SampEn** | | | | | | | | | | | | |
| Memory recognition | Delta | — | 15 | 20 | 0.064 ± 0.036 | 0.105 ± 0.022 | 0.030 | [-0.007, 0.067] | 2.809 | 0.104 | 0.083 | 0.173 |
| Memory recognition | Theta | — | 15 | 20 | 0.141 ± 0.056 | 0.162 ± 0.045 | 0.007 | [-0.058, 0.072] | 0.048 | 0.827 | 0.002 | 0.827 |
| Memory recognition | Alpha | — | 15 | 20 | 0.121 ± 0.044 | 0.170 ± 0.040 | 0.047 | [-0.008, 0.102] | 3.094 | 0.088 | 0.091 | 0.173 |
| Memory recognition | Beta | — | 15 | 20 | 0.460 ± 0.173 | 0.590 ± 0.074 | 0.140 | [-0.022, 0.303] | 3.088 | 0.089 | 0.091 | 0.173 |
| Memory recognition | Gamma | — | 15 | 20 | 0.327 ± 0.133 | 0.412 ± 0.058 | 0.089 | [-0.038, 0.216] | 2.059 | 0.161 | 0.062 | 0.202 |
| **CondEn** | | | | | | | | | | | | |
| Memory recognition | Delta | — | 15 | 20 | 0.100 ± 0.055 | 0.143 ± 0.026 | 0.013 | [-0.038, 0.064] | 0.267 | 0.609 | 0.009 | 0.762 |
| Memory recognition | Theta | — | 15 | 20 | 0.123 ± 0.073 | 0.179 ± 0.039 | 0.026 | [-0.046, 0.097] | 0.542 | 0.467 | 0.017 | 0.762 |
| Memory recognition | Alpha | — | 15 | 20 | 0.102 ± 0.057 | 0.190 ± 0.033 | 0.087 | [0.028, 0.145] | 9.165 | 0.005 | 0.228 | **0.012** |
| Memory recognition | Beta | — | 15 | 20 | 0.584 ± 0.233 | 0.474 ± 0.105 | -0.024 | [-0.244, 0.197] | 0.047 | 0.829 | 0.002 | 0.829 |
| Memory recognition | Gamma | — | 15 | 20 | 0.151 ± 0.097 | 0.317 ± 0.059 | 0.187 | [0.096, 0.278] | 17.668 | <0.001 | 0.363 | **0.001** |

Note. Values are reported as mean ± SD. B is the adjusted difference between older and younger adults from ANCOVA with sex and years of education as covariates. The 95% CI refers to B. F, p and partial η² refer to the group effect. FDR-adjusted p values are reported; values <0.05 are shown in bold. —, not applicable.

**Supplementary Table 7 fNIRS Entropy Parameters in the Auditory Attention Task**

| **Task/Condition** | **Band** | **Hb** | **N young** | **N old** | **Young mean ± SD** | **Old mean ± SD** | **B (old vs. young)** | **95% CI** | **F** | **p** | **partial η²** | **FDR-adjusted p** |
| --- | --- | --- | --- | --- | --- | --- | --- | --- | --- | --- | --- | --- |
| **SampEn** | | | | | | | | | | | | |
| Auditory attention | — | HbO | 14 | 19 | 0.062 ± 0.019 | 0.051 ± 0.019 | -0.007 | [-0.033, 0.020] | 0.291 | 0.594 | 0.010 | 0.594 |
| Auditory attention | — | HbR | 14 | 19 | 0.063 ± 0.018 | 0.054 ± 0.025 | -0.018 | [-0.048, 0.012] | 1.520 | 0.227 | 0.050 | 0.455 |
| **CondEn** | | | | | | | | | | | | |
| Auditory attention | — | HbO | 14 | 19 | 0.145 ± 0.026 | 0.121 ± 0.022 | -0.025 | [-0.057, 0.007] | 2.480 | 0.126 | 0.079 | 0.252 |
| Auditory attention | — | HbR | 14 | 19 | 0.149 ± 0.027 | 0.132 ± 0.038 | -0.016 | [-0.062, 0.030] | 0.530 | 0.473 | 0.018 | 0.473 |

Note. Values are reported as mean ± SD. B is the adjusted difference between older and younger adults from ANCOVA with sex and years of education as covariates. The 95% CI refers to B. F, p and partial η² refer to the group effect. FDR-adjusted p values are reported; values <0.05 are shown in bold. —, not applicable.

**Supplementary Table 8 fNIRS Entropy Parameters in the Verbal Fluency Task**

| **Task/Condition** | **Band** | **Hb** | **N young** | **N old** | **Young mean ± SD** | **Old mean ± SD** | **B (old vs. young)** | **95% CI** | **F** | **p** | **partial η²** | **FDR-adjusted p** |
| --- | --- | --- | --- | --- | --- | --- | --- | --- | --- | --- | --- | --- |
| **SampEn** | | | | | | | | | | | | |
| Verbal fluency | — | HbO | 16 | 19 | 0.070 ± 0.028 | 0.075 ± 0.026 | -0.033 | [-0.065, -0.001] | 4.322 | 0.046 | 0.122 | 0.092 |
| Verbal fluency | — | HbR | 16 | 19 | 0.071 ± 0.028 | 0.066 ± 0.025 | -0.009 | [-0.044, 0.027] | 0.259 | 0.614 | 0.008 | 0.614 |
| **CondEn** | | | | | | | | | | | | |
| Verbal fluency | — | HbO | 16 | 19 | 0.164 ± 0.024 | 0.170 ± 0.029 | -0.025 | [-0.058, 0.008] | 2.432 | 0.129 | 0.073 | 0.258 |
| Verbal fluency | — | HbR | 16 | 19 | 0.172 ± 0.029 | 0.163 ± 0.030 | -0.016 | [-0.055, 0.023] | 0.709 | 0.406 | 0.022 | 0.406 |

Note. Values are reported as mean ± SD. B is the adjusted difference between older and younger adults from ANCOVA with sex and years of education as covariates. The 95% CI refers to B. F, p and partial η² refer to the group effect. FDR-adjusted p values are reported; values <0.05 are shown in bold. —, not applicable.

**Supplementary Table 9 fNIRS Entropy Parameters in the Left Upper Limb Motor Task**

| **Task/Condition** | **Band** | **Hb** | **N young** | **N old** | **Young mean ± SD** | **Old mean ± SD** | **B (old vs. young)** | **95% CI** | **F** | **p** | **partial η²** | **FDR-adjusted p** |
| --- | --- | --- | --- | --- | --- | --- | --- | --- | --- | --- | --- | --- |
| **SampEn** | | | | | | | | | | | | |
| Left motor | — | HbO | 12 | 20 | 0.085 ± 0.023 | 0.066 ± 0.024 | -0.029 | [-0.060, 0.003] | 3.524 | 0.071 | 0.112 | 0.142 |
| Left motor | — | HbR | 12 | 20 | 0.088 ± 0.031 | 0.069 ± 0.016 | -0.017 | [-0.048, 0.013] | 1.385 | 0.249 | 0.047 | 0.249 |
| **CondEn** | | | | | | | | | | | | |
| Left motor | — | HbO | 12 | 20 | 0.183 ± 0.036 | 0.168 ± 0.029 | -0.036 | [-0.077, 0.006] | 3.151 | 0.087 | 0.101 | 0.173 |
| Left motor | — | HbR | 12 | 20 | 0.194 ± 0.037 | 0.169 ± 0.027 | -0.021 | [-0.062, 0.019] | 1.169 | 0.289 | 0.040 | 0.289 |

Note. Values are reported as mean ± SD. B is the adjusted difference between older and younger adults from ANCOVA with sex and years of education as covariates. The 95% CI refers to B. F, p and partial η² refer to the group effect. FDR-adjusted p values are reported; values <0.05 are shown in bold. —, not applicable.

**Supplementary Table 10 fNIRS Entropy Parameters in the Right Upper Limb Motor Task**

| **Task/Condition** | **Band** | **Hb** | **N young** | **N old** | **Young mean ± SD** | **Old mean ± SD** | **B (old vs. young)** | **95% CI** | **F** | **p** | **partial η²** | **FDR-adjusted p** |
| --- | --- | --- | --- | --- | --- | --- | --- | --- | --- | --- | --- | --- |
| **SampEn** | | | | | | | | | | | | |
| Right motor | — | HbO | 12 | 20 | 0.087 ± 0.042 | 0.075 ± 0.026 | -0.008 | [-0.052, 0.037] | 0.120 | 0.732 | 0.004 | 0.732 |
| Right motor | — | HbR | 12 | 20 | 0.092 ± 0.036 | 0.066 ± 0.018 | -0.037 | [-0.071, -0.003] | 4.832 | 0.036 | 0.147 | 0.073 |
| **CondEn** | | | | | | | | | | | | |
| Right motor | — | HbO | 12 | 20 | 0.190 ± 0.053 | 0.170 ± 0.037 | -0.016 | [-0.075, 0.044] | 0.288 | 0.596 | 0.010 | 0.596 |
| Right motor | — | HbR | 12 | 20 | 0.190 ± 0.046 | 0.158 ± 0.026 | -0.031 | [-0.078, 0.015] | 1.897 | 0.179 | 0.063 | 0.359 |

Note. Values are reported as mean ± SD. B is the adjusted difference between older and younger adults from ANCOVA with sex and years of education as covariates. The 95% CI refers to B. F, p and partial η² refer to the group effect. FDR-adjusted p values are reported; values <0.05 are shown in bold. —, not applicable.

**Supplementary Table 11 fNIRS Entropy Parameters in the Encoding Phase of the Recognition Memory Task**

| **Task/Condition** | **Band** | **Hb** | **N young** | **N old** | **Young mean ± SD** | **Old mean ± SD** | **B (old vs. young)** | **95% CI** | **F** | **p** | **partial η²** | **FDR-adjusted p** |
| --- | --- | --- | --- | --- | --- | --- | --- | --- | --- | --- | --- | --- |
| **SampEn** | | | | | | | | | | | | |
| Memory encoding | — | HbO | 15 | 20 | 0.073 ± 0.021 | 0.078 ± 0.035 | 0.004 | [-0.034, 0.042] | 0.044 | 0.835 | 0.001 | 0.835 |
| Memory encoding | — | HbR | 15 | 20 | 0.059 ± 0.019 | 0.073 ± 0.033 | 0.019 | [-0.017, 0.055] | 1.196 | 0.283 | 0.037 | 0.565 |
| **CondEn** | | | | | | | | | | | | |
| Memory encoding | — | HbO | 15 | 20 | 0.165 ± 0.032 | 0.170 ± 0.039 | -0.006 | [-0.053, 0.040] | 0.074 | 0.787 | 0.002 | 0.787 |
| Memory encoding | — | HbR | 15 | 20 | 0.152 ± 0.032 | 0.163 ± 0.032 | 0.018 | [-0.023, 0.060] | 0.810 | 0.375 | 0.025 | 0.750 |

Note. Values are reported as mean ± SD. B is the adjusted difference between older and younger adults from ANCOVA with sex and years of education as covariates. The 95% CI refers to B. F, p and partial η² refer to the group effect. FDR-adjusted p values are reported; values <0.05 are shown in bold. —, not applicable.

**Supplementary Table 12 fNIRS Entropy Parameters in the Recognition Phase of the Recognition Memory Task**

| **Task/Condition** | **Band** | **Hb** | **N young** | **N old** | **Young mean ± SD** | **Old mean ± SD** | **B (old vs. young)** | **95% CI** | **F** | **p** | **partial η²** | **FDR-adjusted p** |
| --- | --- | --- | --- | --- | --- | --- | --- | --- | --- | --- | --- | --- |
| **SampEn** | | | | | | | | | | | | |
| Memory recognition | — | HbO | 15 | 20 | 0.097 ± 0.032 | 0.065 ± 0.022 | -0.040 | [-0.074, -0.006] | 5.800 | 0.022 | 0.158 | **0.044** |
| Memory recognition | — | HbR | 15 | 20 | 0.083 ± 0.026 | 0.062 ± 0.025 | -0.021 | [-0.054, 0.011] | 1.763 | 0.194 | 0.054 | 0.194 |
| **CondEn** | | | | | | | | | | | | |
| Memory recognition | — | HbO | 15 | 20 | 0.179 ± 0.041 | 0.154 ± 0.033 | -0.025 | [-0.070, 0.020] | 1.257 | 0.271 | 0.039 | 0.271 |
| Memory recognition | — | HbR | 15 | 20 | 0.178 ± 0.043 | 0.147 ± 0.037 | -0.032 | [-0.084, 0.020] | 1.597 | 0.216 | 0.049 | 0.271 |

Note. Values are reported as mean ± SD. B is the adjusted difference between older and younger adults from ANCOVA with sex and years of education as covariates. The 95% CI refers to B. F, p and partial η² refer to the group effect. FDR-adjusted p values are reported; values <0.05 are shown in bold. —, not applicable.

**Supplementary Table 13 Cross-Entropy Parameters in the Auditory Attention Task**

| **Task/Condition** | **Band** | **Hb** | **N young** | **N old** | **Young mean ± SD** | **Old mean ± SD** | **B (old vs. young)** | **95% CI** | **F** | **p** | **partial η²** | **FDR-adjusted p** |
| --- | --- | --- | --- | --- | --- | --- | --- | --- | --- | --- | --- | --- |
| **Cross-SampEn** | | | | | | | | | | | | |
| Auditory attention | Delta | HbO | 14 | 19 | 0.130 ± 0.029 | 0.132 ± 0.038 | -0.025 | [-0.069, 0.020] | 1.283 | 0.267 | 0.042 | 0.662 |
| Auditory attention | Theta | HbO | 14 | 19 | 0.125 ± 0.029 | 0.124 ± 0.036 | -0.022 | [-0.067, 0.022] | 1.067 | 0.310 | 0.035 | 0.662 |
| Auditory attention | Alpha | HbO | 14 | 19 | 0.148 ± 0.029 | 0.138 ± 0.029 | -0.004 | [-0.044, 0.035] | 0.049 | 0.826 | 0.002 | 0.826 |
| Auditory attention | Beta | HbO | 14 | 19 | 0.119 ± 0.046 | 0.098 ± 0.020 | -0.005 | [-0.050, 0.039] | 0.063 | 0.803 | 0.002 | 0.826 |
| Auditory attention | Gamma | HbO | 14 | 19 | 0.138 ± 0.027 | 0.113 ± 0.037 | -0.019 | [-0.064, 0.026] | 0.739 | 0.397 | 0.025 | 0.662 |
| Auditory attention | Delta | HbR | 14 | 19 | 0.119 ± 0.022 | 0.136 ± 0.031 | -0.007 | [-0.044, 0.029] | 0.159 | 0.693 | 0.005 | 0.826 |
| Auditory attention | Theta | HbR | 14 | 19 | 0.118 ± 0.024 | 0.129 ± 0.027 | -0.010 | [-0.044, 0.023] | 0.400 | 0.532 | 0.014 | 0.760 |
| Auditory attention | Alpha | HbR | 14 | 19 | 0.144 ± 0.023 | 0.135 ± 0.024 | -0.015 | [-0.047, 0.016] | 0.966 | 0.334 | 0.032 | 0.662 |
| Auditory attention | Beta | HbR | 14 | 19 | 0.116 ± 0.026 | 0.097 ± 0.023 | -0.015 | [-0.048, 0.018] | 0.839 | 0.367 | 0.028 | 0.662 |
| Auditory attention | Gamma | HbR | 14 | 19 | 0.140 ± 0.032 | 0.115 ± 0.038 | -0.032 | [-0.080, 0.016] | 1.894 | 0.179 | 0.061 | 0.662 |
| **Cross-CondEn** | | | | | | | | | | | | |
| Auditory attention | Delta | HbO | 14 | 19 | 1.037 ± 0.145 | 1.146 ± 0.098 | 0.113 | [-0.051, 0.278] | 1.979 | 0.170 | 0.064 | 0.784 |
| Auditory attention | Theta | HbO | 14 | 19 | 1.058 ± 0.115 | 1.143 ± 0.162 | 0.004 | [-0.190, 0.198] | 0.002 | 0.969 | 0.000 | 0.969 |
| Auditory attention | Alpha | HbO | 14 | 19 | 1.179 ± 0.098 | 1.162 ± 0.128 | 0.023 | [-0.133, 0.179] | 0.092 | 0.764 | 0.003 | 0.954 |
| Auditory attention | Beta | HbO | 14 | 19 | 1.054 ± 0.185 | 0.907 ± 0.135 | -0.113 | [-0.326, 0.100] | 1.173 | 0.288 | 0.039 | 0.784 |
| Auditory attention | Gamma | HbO | 14 | 19 | 1.177 ± 0.134 | 1.025 ± 0.128 | -0.071 | [-0.232, 0.090] | 0.820 | 0.373 | 0.027 | 0.784 |
| Auditory attention | Delta | HbR | 14 | 19 | 1.035 ± 0.253 | 1.132 ± 0.151 | 0.116 | [-0.156, 0.387] | 0.756 | 0.392 | 0.025 | 0.784 |
| Auditory attention | Theta | HbR | 14 | 19 | 1.050 ± 0.244 | 1.123 ± 0.127 | -0.016 | [-0.262, 0.230] | 0.017 | 0.896 | 0.001 | 0.969 |
| Auditory attention | Alpha | HbR | 14 | 19 | 1.141 ± 0.147 | 1.149 ± 0.107 | -0.041 | [-0.210, 0.128] | 0.251 | 0.620 | 0.009 | 0.886 |
| Auditory attention | Beta | HbR | 14 | 19 | 0.991 ± 0.168 | 0.966 ± 0.114 | 0.060 | [-0.126, 0.247] | 0.441 | 0.512 | 0.015 | 0.853 |
| Auditory attention | Gamma | HbR | 14 | 19 | 1.200 ± 0.113 | 1.020 ± 0.144 | -0.115 | [-0.266, 0.036] | 2.421 | 0.131 | 0.077 | 0.784 |

Note. Values are reported as mean ± SD. B is the adjusted difference between older and younger adults from ANCOVA with sex and years of education as covariates. The 95% CI refers to B. F, p and partial η² refer to the group effect. FDR-adjusted p values are reported; values <0.05 are shown in bold. —, not applicable.

**Supplementary Table 14 Cross-Entropy Parameters in the Verbal Fluency Task**

| **Task/Condition** | **Band** | **Hb** | **N young** | **N old** | **Young mean ± SD** | **Old mean ± SD** | **B (old vs. young)** | **95% CI** | **F** | **p** | **partial η²** | **FDR-adjusted p** |
| --- | --- | --- | --- | --- | --- | --- | --- | --- | --- | --- | --- | --- |
| **Cross-SampEn** | | | | | | | | | | | | |
| Verbal fluency | Delta | HbO | 16 | 19 | 0.162 ± 0.044 | 0.183 ± 0.040 | 0.026 | [-0.029, 0.082] | 0.944 | 0.339 | 0.030 | 0.678 |
| Verbal fluency | Theta | HbO | 16 | 19 | 0.164 ± 0.051 | 0.174 ± 0.036 | -0.017 | [-0.073, 0.039] | 0.387 | 0.539 | 0.012 | 0.769 |
| Verbal fluency | Alpha | HbO | 16 | 19 | 0.174 ± 0.055 | 0.183 ± 0.042 | -0.005 | [-0.067, 0.056] | 0.032 | 0.859 | 0.001 | 0.955 |
| Verbal fluency | Beta | HbO | 16 | 19 | 0.157 ± 0.050 | 0.149 ± 0.032 | -0.033 | [-0.086, 0.021] | 1.576 | 0.219 | 0.048 | 0.678 |
| Verbal fluency | Gamma | HbO | 16 | 19 | 0.167 ± 0.041 | 0.178 ± 0.033 | 0.048 | [0.002, 0.095] | 4.474 | 0.043 | 0.126 | 0.426 |
| Verbal fluency | Delta | HbR | 16 | 19 | 0.163 ± 0.036 | 0.168 ± 0.036 | 0.007 | [-0.041, 0.055] | 0.090 | 0.767 | 0.003 | 0.955 |
| Verbal fluency | Theta | HbR | 16 | 19 | 0.160 ± 0.054 | 0.169 ± 0.039 | 0.029 | [-0.031, 0.089] | 0.963 | 0.334 | 0.030 | 0.678 |
| Verbal fluency | Alpha | HbR | 16 | 19 | 0.168 ± 0.040 | 0.174 ± 0.024 | 0.014 | [-0.029, 0.057] | 0.420 | 0.521 | 0.013 | 0.769 |
| Verbal fluency | Beta | HbR | 16 | 19 | 0.154 ± 0.052 | 0.129 ± 0.035 | -0.043 | [-0.101, 0.014] | 2.340 | 0.136 | 0.070 | 0.678 |
| Verbal fluency | Gamma | HbR | 16 | 19 | 0.175 ± 0.052 | 0.169 ± 0.037 | 0.001 | [-0.058, 0.060] | 0.001 | 0.970 | 0.000 | 0.970 |
| **Cross-CondEn** | | | | | | | | | | | | |
| Verbal fluency | Delta | HbO | 16 | 19 | 1.037 ± 0.185 | 1.021 ± 0.164 | 0.000 | [-0.232, 0.231] | 0.000 | 0.997 | 0.000 | 0.997 |
| Verbal fluency | Theta | HbO | 16 | 19 | 1.084 ± 0.167 | 1.104 ± 0.158 | -0.024 | [-0.238, 0.191] | 0.050 | 0.824 | 0.002 | 0.915 |
| Verbal fluency | Alpha | HbO | 16 | 19 | 1.130 ± 0.142 | 1.028 ± 0.165 | -0.040 | [-0.243, 0.164] | 0.161 | 0.691 | 0.005 | 0.915 |
| Verbal fluency | Beta | HbO | 16 | 19 | 1.047 ± 0.193 | 1.078 ± 0.216 | 0.103 | [-0.159, 0.366] | 0.647 | 0.427 | 0.020 | 0.915 |
| Verbal fluency | Gamma | HbO | 16 | 19 | 1.078 ± 0.150 | 1.088 ± 0.131 | 0.037 | [-0.149, 0.224] | 0.167 | 0.686 | 0.005 | 0.915 |
| Verbal fluency | Delta | HbR | 16 | 19 | 0.943 ± 0.209 | 1.061 ± 0.138 | 0.180 | [-0.050, 0.410] | 2.538 | 0.121 | 0.076 | 0.606 |
| Verbal fluency | Theta | HbR | 16 | 19 | 1.021 ± 0.192 | 1.082 ± 0.110 | -0.048 | [-0.240, 0.145] | 0.255 | 0.617 | 0.008 | 0.915 |
| Verbal fluency | Alpha | HbR | 16 | 19 | 1.080 ± 0.131 | 1.072 ± 0.164 | 0.031 | [-0.165, 0.226] | 0.102 | 0.752 | 0.003 | 0.915 |
| Verbal fluency | Beta | HbR | 16 | 19 | 0.989 ± 0.175 | 1.070 ± 0.156 | 0.050 | [-0.169, 0.269] | 0.216 | 0.645 | 0.007 | 0.915 |
| Verbal fluency | Gamma | HbR | 16 | 19 | 1.033 ± 0.129 | 1.097 ± 0.117 | 0.138 | [-0.013, 0.289] | 3.491 | 0.071 | 0.101 | 0.606 |

Note. Values are reported as mean ± SD. B is the adjusted difference between older and younger adults from ANCOVA with sex and years of education as covariates. The 95% CI refers to B. F, p and partial η² refer to the group effect. FDR-adjusted p values are reported; values <0.05 are shown in bold. —, not applicable.

**Supplementary Table 15 Cross-Entropy Parameters in the Left Upper Limb Motor Task**

| **Task/Condition** | **Band** | **Hb** | **N young** | **N old** | **Young mean ± SD** | **Old mean ± SD** | **B (old vs. young)** | **95% CI** | **F** | **p** | **partial η²** | **FDR-adjusted p** |
| --- | --- | --- | --- | --- | --- | --- | --- | --- | --- | --- | --- | --- |
| **Cross-SampEn** | | | | | | | | | | | | |
| Left motor | Delta | HbO | 12 | 20 | 0.172 ± 0.039 | 0.184 ± 0.026 | 0.006 | [-0.036, 0.049] | 0.093 | 0.763 | 0.003 | 0.848 |
| Left motor | Theta | HbO | 12 | 20 | 0.188 ± 0.045 | 0.179 ± 0.042 | -0.025 | [-0.083, 0.034] | 0.757 | 0.392 | 0.026 | 0.814 |
| Left motor | Alpha | HbO | 12 | 20 | 0.193 ± 0.041 | 0.173 ± 0.037 | -0.015 | [-0.067, 0.038] | 0.331 | 0.570 | 0.012 | 0.814 |
| Left motor | Beta | HbO | 12 | 20 | 0.173 ± 0.037 | 0.143 ± 0.033 | -0.045 | [-0.091, 0.002] | 3.878 | 0.059 | 0.122 | 0.294 |
| Left motor | Gamma | HbO | 12 | 20 | 0.196 ± 0.039 | 0.176 ± 0.041 | -0.027 | [-0.081, 0.028] | 1.010 | 0.324 | 0.035 | 0.814 |
| Left motor | Delta | HbR | 12 | 20 | 0.180 ± 0.036 | 0.183 ± 0.030 | 0.015 | [-0.028, 0.059] | 0.520 | 0.477 | 0.018 | 0.814 |
| Left motor | Theta | HbR | 12 | 20 | 0.158 ± 0.045 | 0.184 ± 0.031 | 0.017 | [-0.033, 0.067] | 0.478 | 0.495 | 0.017 | 0.814 |
| Left motor | Alpha | HbR | 12 | 20 | 0.186 ± 0.045 | 0.171 ± 0.032 | -0.009 | [-0.058, 0.041] | 0.125 | 0.726 | 0.004 | 0.848 |
| Left motor | Beta | HbR | 12 | 20 | 0.172 ± 0.039 | 0.136 ± 0.030 | -0.070 | [-0.113, -0.027] | 11.051 | 0.002 | 0.283 | **0.025** |
| Left motor | Gamma | HbR | 12 | 20 | 0.179 ± 0.050 | 0.180 ± 0.034 | -0.005 | [-0.061, 0.050] | 0.036 | 0.851 | 0.001 | 0.851 |
| **Cross-CondEn** | | | | | | | | | | | | |
| Left motor | Delta | HbO | 12 | 20 | 1.126 ± 0.117 | 1.076 ± 0.186 | -0.168 | [-0.385, 0.049] | 2.519 | 0.124 | 0.083 | 0.412 |
| Left motor | Theta | HbO | 12 | 20 | 1.081 ± 0.181 | 1.047 ± 0.127 | -0.154 | [-0.346, 0.038] | 2.690 | 0.112 | 0.088 | 0.412 |
| Left motor | Alpha | HbO | 12 | 20 | 1.102 ± 0.243 | 1.031 ± 0.171 | 0.010 | [-0.263, 0.283] | 0.005 | 0.942 | 0.000 | 0.983 |
| Left motor | Beta | HbO | 12 | 20 | 1.112 ± 0.122 | 1.119 ± 0.114 | 0.002 | [-0.158, 0.162] | 0.000 | 0.983 | 0.000 | 0.983 |
| Left motor | Gamma | HbO | 12 | 20 | 1.085 ± 0.139 | 1.076 ± 0.151 | -0.125 | [-0.310, 0.060] | 1.920 | 0.177 | 0.064 | 0.442 |
| Left motor | Delta | HbR | 12 | 20 | 1.112 ± 0.190 | 1.092 ± 0.080 | -0.062 | [-0.242, 0.118] | 0.495 | 0.488 | 0.017 | 0.813 |
| Left motor | Theta | HbR | 12 | 20 | 1.014 ± 0.239 | 1.060 ± 0.106 | -0.014 | [-0.241, 0.213] | 0.016 | 0.899 | 0.001 | 0.983 |
| Left motor | Alpha | HbR | 12 | 20 | 1.110 ± 0.150 | 1.087 ± 0.148 | -0.045 | [-0.248, 0.158] | 0.205 | 0.654 | 0.007 | 0.934 |
| Left motor | Beta | HbR | 12 | 20 | 1.062 ± 0.211 | 1.015 ± 0.204 | -0.260 | [-0.523, 0.004] | 4.064 | 0.054 | 0.127 | 0.412 |
| Left motor | Gamma | HbR | 12 | 20 | 1.108 ± 0.155 | 1.101 ± 0.152 | -0.104 | [-0.309, 0.101] | 1.072 | 0.309 | 0.037 | 0.619 |

Note. Values are reported as mean ± SD. B is the adjusted difference between older and younger adults from ANCOVA with sex and years of education as covariates. The 95% CI refers to B. F, p and partial η² refer to the group effect. FDR-adjusted p values are reported; values <0.05 are shown in bold. —, not applicable.

**Supplementary Table 16 Cross-Entropy Parameters in the Right Upper Limb Motor Task**

| **Task/Condition** | **Band** | **Hb** | **N young** | **N old** | **Young mean ± SD** | **Old mean ± SD** | **B (old vs. young)** | **95% CI** | **F** | **p** | **partial η²** | **FDR-adjusted p** |
| --- | --- | --- | --- | --- | --- | --- | --- | --- | --- | --- | --- | --- |
| **Cross-SampEn** | | | | | | | | | | | | |
| Right motor | Delta | HbO | 12 | 20 | 0.191 ± 0.055 | 0.195 ± 0.040 | -0.019 | [-0.080, 0.041] | 0.420 | 0.522 | 0.015 | 0.771 |
| Right motor | Theta | HbO | 12 | 20 | 0.201 ± 0.049 | 0.182 ± 0.036 | -0.037 | [-0.094, 0.019] | 1.826 | 0.187 | 0.061 | 0.415 |
| Right motor | Alpha | HbO | 12 | 20 | 0.187 ± 0.033 | 0.196 ± 0.045 | -0.033 | [-0.085, 0.019] | 1.664 | 0.208 | 0.056 | 0.415 |
| Right motor | Beta | HbO | 12 | 20 | 0.171 ± 0.068 | 0.154 ± 0.031 | -0.048 | [-0.111, 0.016] | 2.387 | 0.134 | 0.079 | 0.415 |
| Right motor | Gamma | HbO | 12 | 20 | 0.162 ± 0.032 | 0.177 ± 0.039 | -0.010 | [-0.059, 0.038] | 0.191 | 0.665 | 0.007 | 0.832 |
| Right motor | Delta | HbR | 12 | 20 | 0.179 ± 0.037 | 0.190 ± 0.048 | 0.042 | [-0.017, 0.101] | 2.156 | 0.153 | 0.071 | 0.415 |
| Right motor | Theta | HbR | 12 | 20 | 0.190 ± 0.039 | 0.164 ± 0.032 | -0.005 | [-0.051, 0.042] | 0.040 | 0.843 | 0.001 | 0.937 |
| Right motor | Alpha | HbR | 12 | 20 | 0.186 ± 0.033 | 0.188 ± 0.040 | 0.015 | [-0.036, 0.067] | 0.386 | 0.539 | 0.014 | 0.771 |
| Right motor | Beta | HbR | 12 | 20 | 0.158 ± 0.040 | 0.143 ± 0.033 | 0.030 | [-0.014, 0.073] | 1.942 | 0.174 | 0.065 | 0.415 |
| Right motor | Gamma | HbR | 12 | 20 | 0.182 ± 0.036 | 0.188 ± 0.033 | 0.001 | [-0.046, 0.047] | 0.001 | 0.981 | 0.000 | 0.981 |
| **Cross-CondEn** | | | | | | | | | | | | |
| Right motor | Delta | HbO | 12 | 20 | 1.046 ± 0.134 | 1.094 ± 0.159 | -0.004 | [-0.208, 0.200] | 0.002 | 0.968 | 0.000 | 0.968 |
| Right motor | Theta | HbO | 12 | 20 | 1.143 ± 0.087 | 1.079 ± 0.146 | -0.126 | [-0.299, 0.047] | 2.219 | 0.147 | 0.073 | 0.737 |
| Right motor | Alpha | HbO | 12 | 20 | 1.127 ± 0.190 | 1.069 ± 0.148 | -0.043 | [-0.253, 0.167] | 0.176 | 0.678 | 0.006 | 0.968 |
| Right motor | Beta | HbO | 12 | 20 | 1.075 ± 0.177 | 1.044 ± 0.145 | 0.011 | [-0.204, 0.227] | 0.012 | 0.915 | 0.000 | 0.968 |
| Right motor | Gamma | HbO | 12 | 20 | 1.075 ± 0.153 | 1.093 ± 0.148 | 0.156 | [-0.039, 0.350] | 2.685 | 0.112 | 0.087 | 0.737 |
| Right motor | Delta | HbR | 12 | 20 | 1.044 ± 0.156 | 1.123 ± 0.150 | 0.045 | [-0.154, 0.243] | 0.211 | 0.649 | 0.007 | 0.968 |
| Right motor | Theta | HbR | 12 | 20 | 1.105 ± 0.136 | 1.136 ± 0.162 | -0.072 | [-0.274, 0.131] | 0.525 | 0.475 | 0.018 | 0.968 |
| Right motor | Alpha | HbR | 12 | 20 | 1.181 ± 0.137 | 1.123 ± 0.153 | -0.021 | [-0.212, 0.169] | 0.053 | 0.820 | 0.002 | 0.968 |
| Right motor | Beta | HbR | 12 | 20 | 1.127 ± 0.137 | 1.003 ± 0.168 | -0.029 | [-0.238, 0.181] | 0.077 | 0.783 | 0.003 | 0.968 |
| Right motor | Gamma | HbR | 12 | 20 | 1.098 ± 0.168 | 1.027 ± 0.142 | 0.087 | [-0.100, 0.274] | 0.911 | 0.348 | 0.032 | 0.968 |

Note. Values are reported as mean ± SD. B is the adjusted difference between older and younger adults from ANCOVA with sex and years of education as covariates. The 95% CI refers to B. F, p and partial η² refer to the group effect. FDR-adjusted p values are reported; values <0.05 are shown in bold. —, not applicable.

**Supplementary Table 17 Cross-Entropy Parameters in the Encoding Phase of the Recognition Memory Task**

| **Task/Condition** | **Band** | **Hb** | **N young** | **N old** | **Young mean ± SD** | **Old mean ± SD** | **B (old vs. young)** | **95% CI** | **F** | **p** | **partial η²** | **FDR-adjusted p** |
| --- | --- | --- | --- | --- | --- | --- | --- | --- | --- | --- | --- | --- |
| **Cross-SampEn** | | | | | | | | | | | | |
| Memory encoding | Delta | HbO | 15 | 20 | 0.156 ± 0.030 | 0.151 ± 0.031 | -0.016 | [-0.055, 0.024] | 0.661 | 0.422 | 0.021 | 0.703 |
| Memory encoding | Theta | HbO | 15 | 20 | 0.134 ± 0.030 | 0.147 ± 0.022 | 0.020 | [-0.013, 0.054] | 1.527 | 0.226 | 0.047 | 0.564 |
| Memory encoding | Alpha | HbO | 15 | 20 | 0.142 ± 0.032 | 0.163 ± 0.030 | 0.042 | [0.003, 0.081] | 4.853 | 0.035 | 0.135 | 0.176 |
| Memory encoding | Beta | HbO | 15 | 20 | 0.125 ± 0.038 | 0.129 ± 0.034 | -0.014 | [-0.059, 0.031] | 0.391 | 0.536 | 0.012 | 0.703 |
| Memory encoding | Gamma | HbO | 15 | 20 | 0.152 ± 0.029 | 0.149 ± 0.028 | -0.003 | [-0.039, 0.033] | 0.026 | 0.873 | 0.001 | 0.873 |
| Memory encoding | Delta | HbR | 15 | 20 | 0.151 ± 0.023 | 0.163 ± 0.034 | 0.036 | [-0.002, 0.073] | 3.822 | 0.060 | 0.110 | 0.199 |
| Memory encoding | Theta | HbR | 15 | 20 | 0.125 ± 0.041 | 0.161 ± 0.037 | 0.072 | [0.025, 0.118] | 9.933 | 0.004 | 0.243 | **0.036** |
| Memory encoding | Alpha | HbR | 15 | 20 | 0.153 ± 0.049 | 0.166 ± 0.032 | 0.027 | [-0.025, 0.079] | 1.106 | 0.301 | 0.034 | 0.602 |
| Memory encoding | Beta | HbR | 15 | 20 | 0.122 ± 0.040 | 0.135 ± 0.029 | 0.012 | [-0.031, 0.055] | 0.343 | 0.562 | 0.011 | 0.703 |
| Memory encoding | Gamma | HbR | 15 | 20 | 0.144 ± 0.035 | 0.142 ± 0.028 | 0.007 | [-0.033, 0.046] | 0.114 | 0.738 | 0.004 | 0.820 |
| **Cross-CondEn** | | | | | | | | | | | | |
| Memory encoding | Delta | HbO | 15 | 20 | 1.180 ± 0.093 | 1.095 ± 0.170 | -0.014 | [-0.193, 0.165] | 0.027 | 0.872 | 0.001 | 0.963 |
| Memory encoding | Theta | HbO | 15 | 20 | 1.106 ± 0.143 | 1.165 ± 0.156 | 0.114 | [-0.081, 0.309] | 1.417 | 0.243 | 0.044 | 0.708 |
| Memory encoding | Alpha | HbO | 15 | 20 | 1.200 ± 0.177 | 1.126 ± 0.115 | -0.020 | [-0.206, 0.166] | 0.047 | 0.829 | 0.002 | 0.963 |
| Memory encoding | Beta | HbO | 15 | 20 | 1.096 ± 0.202 | 1.065 ± 0.149 | -0.133 | [-0.344, 0.078] | 1.653 | 0.208 | 0.051 | 0.708 |
| Memory encoding | Gamma | HbO | 15 | 20 | 1.169 ± 0.178 | 1.128 ± 0.127 | -0.004 | [-0.196, 0.187] | 0.002 | 0.963 | 0.000 | 0.963 |
| Memory encoding | Delta | HbR | 15 | 20 | 1.104 ± 0.162 | 1.062 ± 0.203 | -0.072 | [-0.302, 0.158] | 0.407 | 0.528 | 0.013 | 0.881 |
| Memory encoding | Theta | HbR | 15 | 20 | 0.980 ± 0.204 | 1.155 ± 0.163 | 0.098 | [-0.137, 0.333] | 0.723 | 0.402 | 0.023 | 0.803 |
| Memory encoding | Alpha | HbR | 15 | 20 | 1.114 ± 0.121 | 1.121 ± 0.161 | 0.099 | [-0.086, 0.283] | 1.192 | 0.283 | 0.037 | 0.708 |
| Memory encoding | Beta | HbR | 15 | 20 | 1.038 ± 0.206 | 1.083 ± 0.174 | -0.017 | [-0.260, 0.226] | 0.020 | 0.889 | 0.001 | 0.963 |
| Memory encoding | Gamma | HbR | 15 | 20 | 1.101 ± 0.141 | 1.033 ± 0.163 | -0.117 | [-0.309, 0.074] | 1.557 | 0.221 | 0.048 | 0.708 |

Note. Values are reported as mean ± SD. B is the adjusted difference between older and younger adults from ANCOVA with sex and years of education as covariates. The 95% CI refers to B. F, p and partial η² refer to the group effect. FDR-adjusted p values are reported; values <0.05 are shown in bold. —, not applicable.

**Supplementary Table 18 Cross-Entropy Parameters in the Recognition Phase of the Recognition Memory Task**

| **Task/Condition** | **Band** | **Hb** | **N young** | **N old** | **Young mean ± SD** | **Old mean ± SD** | **B (old vs. young)** | **95% CI** | **F** | **p** | **partial η²** | **FDR-adjusted p** |
| --- | --- | --- | --- | --- | --- | --- | --- | --- | --- | --- | --- | --- |
| **Cross-SampEn** | | | | | | | | | | | | |
| Memory recognition | Delta | HbO | 15 | 20 | 0.167 ± 0.037 | 0.166 ± 0.039 | -0.021 | [-0.068, 0.027] | 0.786 | 0.382 | 0.025 | 0.735 |
| Memory recognition | Theta | HbO | 15 | 20 | 0.147 ± 0.020 | 0.148 ± 0.034 | 0.007 | [-0.029, 0.042] | 0.151 | 0.700 | 0.005 | 0.735 |
| Memory recognition | Alpha | HbO | 15 | 20 | 0.147 ± 0.025 | 0.151 ± 0.037 | 0.007 | [-0.035, 0.049] | 0.117 | 0.735 | 0.004 | 0.735 |
| Memory recognition | Beta | HbO | 15 | 20 | 0.144 ± 0.040 | 0.119 ± 0.027 | -0.009 | [-0.051, 0.033] | 0.182 | 0.672 | 0.006 | 0.735 |
| Memory recognition | Gamma | HbO | 15 | 20 | 0.143 ± 0.021 | 0.159 ± 0.032 | 0.021 | [-0.016, 0.058] | 1.374 | 0.250 | 0.042 | 0.735 |
| Memory recognition | Delta | HbR | 15 | 20 | 0.158 ± 0.036 | 0.169 ± 0.040 | -0.017 | [-0.066, 0.031] | 0.519 | 0.477 | 0.016 | 0.735 |
| Memory recognition | Theta | HbR | 15 | 20 | 0.140 ± 0.036 | 0.148 ± 0.031 | -0.023 | [-0.064, 0.018] | 1.293 | 0.264 | 0.040 | 0.735 |
| Memory recognition | Alpha | HbR | 15 | 20 | 0.138 ± 0.022 | 0.156 ± 0.054 | 0.012 | [-0.045, 0.069] | 0.182 | 0.673 | 0.006 | 0.735 |
| Memory recognition | Beta | HbR | 15 | 20 | 0.130 ± 0.043 | 0.116 ± 0.030 | -0.028 | [-0.074, 0.019] | 1.490 | 0.231 | 0.046 | 0.735 |
| Memory recognition | Gamma | HbR | 15 | 20 | 0.147 ± 0.040 | 0.138 ± 0.040 | -0.014 | [-0.066, 0.037] | 0.325 | 0.573 | 0.010 | 0.735 |
| **Cross-CondEn** | | | | | | | | | | | | |
| Memory recognition | Delta | HbO | 15 | 20 | 1.165 ± 0.171 | 1.178 ± 0.172 | 0.092 | [-0.130, 0.313] | 0.712 | 0.405 | 0.022 | 0.748 |
| Memory recognition | Theta | HbO | 15 | 20 | 1.159 ± 0.075 | 1.165 ± 0.156 | 0.088 | [-0.076, 0.251] | 1.197 | 0.282 | 0.037 | 0.720 |
| Memory recognition | Alpha | HbO | 15 | 20 | 1.109 ± 0.195 | 1.120 ± 0.153 | 0.026 | [-0.196, 0.248] | 0.058 | 0.811 | 0.002 | 0.829 |
| Memory recognition | Beta | HbO | 15 | 20 | 1.117 ± 0.158 | 1.081 ± 0.128 | -0.019 | [-0.197, 0.159] | 0.047 | 0.829 | 0.002 | 0.829 |
| Memory recognition | Gamma | HbO | 15 | 20 | 1.113 ± 0.114 | 1.156 ± 0.155 | 0.165 | [-0.008, 0.338] | 3.793 | 0.061 | 0.109 | 0.303 |
| Memory recognition | Delta | HbR | 15 | 20 | 1.109 ± 0.197 | 1.166 ± 0.207 | -0.063 | [-0.322, 0.196] | 0.246 | 0.624 | 0.008 | 0.780 |
| Memory recognition | Theta | HbR | 15 | 20 | 1.154 ± 0.201 | 1.133 ± 0.133 | -0.067 | [-0.281, 0.147] | 0.409 | 0.527 | 0.013 | 0.753 |
| Memory recognition | Alpha | HbR | 15 | 20 | 1.104 ± 0.200 | 1.069 ± 0.208 | -0.100 | [-0.366, 0.166] | 0.588 | 0.449 | 0.019 | 0.748 |
| Memory recognition | Beta | HbR | 15 | 20 | 1.105 ± 0.152 | 1.014 ± 0.150 | -0.250 | [-0.433, -0.068] | 7.863 | 0.009 | 0.202 | 0.086 |
| Memory recognition | Gamma | HbR | 15 | 20 | 1.180 ± 0.126 | 1.138 ± 0.186 | -0.110 | [-0.317, 0.097] | 1.170 | 0.288 | 0.036 | 0.720 |

Note. Values are reported as mean ± SD. B is the adjusted difference between older and younger adults from ANCOVA with sex and years of education as covariates. The 95% CI refers to B. F, p and partial η² refer to the group effect. FDR-adjusted p values are reported; values <0.05 are shown in bold. —, not applicable.

**Supplementary Table 19 Group × Education Interaction Tests for Primary Significant Outcomes**

| **Task/Condition** | **Metric** | **Band** | **Hb** | **N young** | **N old** | **B** | **95% CI** | **F** | **p** | **partial η²** |
| --- | --- | --- | --- | --- | --- | --- | --- | --- | --- | --- |
| **Auditory attention** | | | | | | | | | | |
| Auditory attention | SampEn | Alpha | — | 14 | 19 | -0.007 | [-0.024, 0.009] | 0.875 | 0.358 | 0.031 |
| Auditory attention | CondEn | Delta | — | 14 | 19 | 0.004 | [-0.018, 0.027] | 0.167 | 0.686 | 0.006 |
| Auditory attention | CondEn | Theta | — | 14 | 19 | -0.005 | [-0.031, 0.020] | 0.193 | 0.664 | 0.007 |
| Auditory attention | CondEn | Gamma | — | 14 | 19 | -0.019 | [-0.064, 0.026] | 0.751 | 0.394 | 0.027 |
| **Left upper limb motor** | | | | | | | | | | |
| Left upper limb motor | Cross-SampEn | Beta | HbR | 12 | 20 | 0.003 | [-0.013, 0.019] | 0.150 | 0.701 | 0.006 |
| **Memory encoding** | | | | | | | | | | |
| Memory encoding | SampEn | Gamma | — | 15 | 20 | 0.017 | [-0.011, 0.046] | 1.525 | 0.227 | 0.050 |
| Memory encoding | CondEn | Alpha | — | 15 | 20 | 0.016 | [-0.006, 0.037] | 2.301 | 0.140 | 0.074 |
| Memory encoding | CondEn | Gamma | — | 15 | 20 | 0.023 | [-0.010, 0.055] | 2.061 | 0.162 | 0.066 |
| Memory encoding | Cross-SampEn | Theta | HbR | 15 | 20 | -0.002 | [-0.019, 0.015] | 0.061 | 0.806 | 0.002 |
| **Memory recognition** | | | | | | | | | | |
| Memory recognition | CondEn | Alpha | — | 15 | 20 | 0.006 | [-0.013, 0.025] | 0.413 | 0.526 | 0.014 |
| Memory recognition | CondEn | Gamma | — | 15 | 20 | 0.024 | [-0.004, 0.052] | 3.029 | 0.092 | 0.095 |
| Memory recognition | SampEn | — | HbO | 15 | 20 | 0.001 | [-0.012, 0.013] | 0.014 | 0.908 | 0.000 |

Note. Group × Education statistics were estimated in ANCOVA models including age group, sex, education, and both Group × Education and Group × Sex interactions. B is the slope difference between older and younger adults per additional education year (young reference). The 95% CI refers to B. F, p, and partial η² refer to Group × Education. Tests are exploratory; p values are unadjusted. —, not applicable.
